# Supplementary material for: Phytophenol Dimerization Reaction: From Basic Rules to Diastereoselectivity and Beyond
Source: Molecules. 2022 Jul 28;27(15):4842. doi: 10.3390/molecules27154842 (PMC9369853; doi:10.3390/molecules27154842)
Supplement: Supplementary file 1 [file molecules-27-04842-s001.zip › Supplementary File S1 Product identification in situ.pdf]

Suppl. 1-Product identification *in situ*

Phytophenol Dimerization Reaction: From Basic Rules to Diastereoselectivity and Beyond

Shuqin Liu [1,†], Xican Li [1,*,†], Ban Chen [1], Xiaojian Ouyang [1], Yulu Xie [1] and Dongfeng Chen [2,*]

[1] School of Chinese Herbal Medicine, Guangzhou University of Chinese Medicine, Guangzhou Higher Education Mega Center, Waihuang East Road No. 232, Guangzhou 510006, China; 20201110616@stu.gzucm.edu.cn (S.L.); imchenban@foxmail.com(B.C.); oyxiaojian55@163.com (X.O.); xieyulu1900@163.com (Y.X.)

[2] School of Basic Medical Science, Guangzhou University of Chinese Medicine, Guangzhou Higher Education Mega Center, Waihuang East Road No. 232, Guangzhou 510006, China

* Correspondence: lixican@126.com (X.L.); chen888@gzucm.edu.cn (D.C.)

† These authors contributed equally to this work.

Homepage http://www.researchgate.net/profile/Xican_Li

Tel: +86-20-39358076 **Fax:** +86-20-38892690

Note:

The dimerization reaction was initiated using DPPH$^{\bullet}$ or enzyme in methanol solution. The product identifications or exclusions were based on the comparison of their MS/MS spectra, a commonly used method for *in situ* identification [1],[2]. To enhance the accuracy of identification, some important MS fragments (especially molecular ion peaks) were also listed their calculated *m/z* values. The *m/z* value calculation is based on the relative atomic masses of C (12.0000), H (1.007825), O (15.994915), and N (14.003074) [3]. The relative standard deviation (RSD) value was also calculated, using the formula: RSD = experimental *m/z* value ÷ calculated *m/z* value.

Contents

Fig. S1.1 The molecular ion peak and MS/MS spectra of standard 2,2'-biphenol (**1a**)
Fig. S1.2 The molecular ion peak and MS/MS spectra of standard 2,4'-biphenol (**1b**)
Fig. S1.3 The molecular ion peak and MS/MS spectra of standard 4,4'-biphenol (**1c**)
Fig. S1.4 The molecular ion peak and MS/MS spectra of standard 4-hydroxyldiphenyl ether (**1d**)
Fig. S1.5 The molecular ion peak and MS/MS spectra of standard 2-hydroxyldiphenyl ether (**1e**)
Fig. S1.6 The main UHPLC–ESI-Q-TOF-MS results of dimerization products of DPPH•-treated phenol (**1**) in methanol and product identifications
Fig. S2.1 The molecular ion peak and MS/MS spectra of standard magnolol (**2a**)
Fig. S2.2 The molecular ion peak and MS/MS spectra of standard 4,4'-diallyl-1-hydroxyldiphenyl ether (**2b**)
Fig. S2.3 The main UHPLC–ESI-Q-TOF-MS results of dimerization products of DPPH•-treated 4-allylphenol (**2**) in methanol and product identifications
Fig. S3.1 The molecular ion peak and MS/MS spectra of standard honokiol (**3a**)
Fig. S3.2 The main UHPLC–ESI-Q-TOF-MS results of products of DPPH•-treated 4-allylphenol (**2**) *plus* 2-allyphenold (**3**) in methanol.
Fig. S4.1 The chromatographic peaks of dimerization products of DPPH•-treated syringic acid (**4**) in methanol
Fig. S5.1 The screenshot of documented data and elucidation of [6]-gingerol 6,6'-dimer (**5a**) and [10]-gingerol 6,6'-dimer (**6a**)
Fig. S5.2 The main UHPLC–ESI-Q-TOF-MS results of dimerization products of DPPH•-treated [6]-gingerol (**5**) in methanol and [6]-gingerol 6,6' dimer (**5a**) identification.
Fig. S6.1 The main UHPLC–ESI-Q-TOF-MS results of dimerization products of DPPH•-treated [10]-gingerol (**6**) in methanol and [10]-gingerol 6,6' dimer (**6a**) identification
Fig. S7.1 The MS elucidation of capsaicin 6,6'-dimer (**7a**)
Fig. S7.2 The main UHPLC–ESI-Q-TOF-MS results of dimerization products of DPPH•-treated capsaicin (**7**) in methanol and product identification.
Fig. S8.1 The molecular ion peak and MS/MS spectra of standard tyrosine 3,3'-dimer (**8a**)
Fig. S8.2 The main UHPLC–ESI-Q-TOF-MS results of dimerization products of DPPH•-treated **tyrosine** (**8**) in methanol and product identification.
Fig. S9.1 The molecular ion peak and MS/MS spectra of standard euphorbetin (**9a**)
Fig. S9.2 The molecular ion peak and MS/MS spectra of standard isoeuphorbetin (**9b**)
Fig. S9.3 The main UHPLC–ESI-Q-TOF-MS results of dimerization products of DPPH•-treated esculetin (**9**) in methanol and product identification
Fig. S10.1 The molecular ion peak and MS/MS spectra of standard 3,6-dimethoxycatechol β,β'-dimer (**10a**)
Fig. S10.2 The main UHPLC–ESI-Q-TOF-MS results of dimerization products of DPPH•-treated 3,6-dimethoxycatechol (**10**) in methanol and product identification
Fig. S11.1 The MS elucidation of bis-eugenol (**11a**)
Fig. S11.2 The main UHPLC–ESI-Q-TOF-MS results of dimerization products of DPPH•-treated eugenol (**11**) in methanol and product identification.
Fig. S12.1 The molecular ion peak and MS/MS spectra of standard dehydrodiisoeugenol (**12a**).
Fig. S12.2 The main UHPLC–ESI-Q-TOF-MS results of dimerization products of DPPH•-treated isoeugenol (**12**) in methanol and product identification.
Fig. S13.1 The typical UHPLC–ESI-Q-TOF-MS results of dimerization products of DPPH•-treated coniferyl alcohol (**13**) in methanol.
Fig. S13.2 The typical UHPLC–ESI-Q-TOF-MS results of dimerization products of HRP-catalyzed coniferyl alcohol (**13**)
Fig. S13.3 The main results of HPLC-UV analyses for product identification of coniferyl alcohol (**13**) dimerization
References

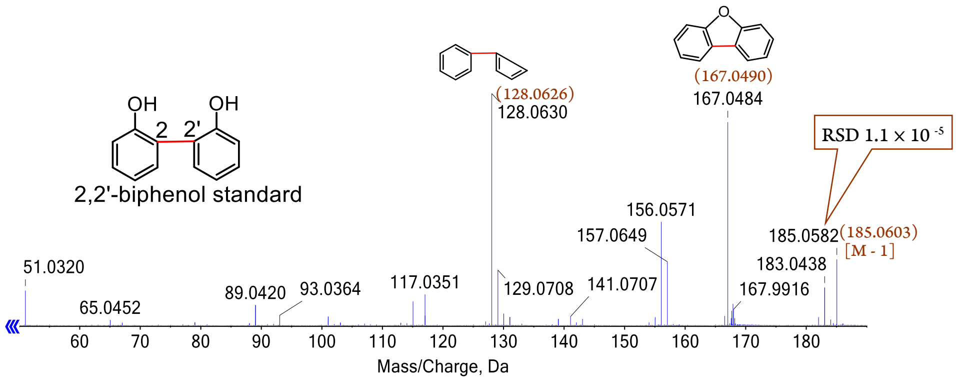

Fig. S1.1 The molecular ion peak and MS/MS spectra of standard 2,2'-biphenol (**1a**, $C_{12}H_{10}O_2$, CAS 1806-29-7, M.W. 186.0681) The bracketed value is the calculated *m/z* value. RSD = experimental *m/z* value ÷ calculated *m/z* value.

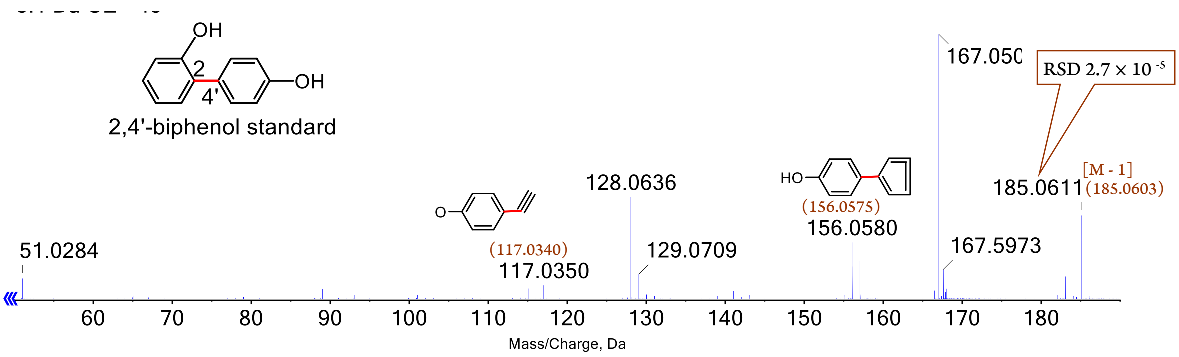

Fig. S1.2 The molecular ion peak and MS/MS spectra of standard 2,4'-biphenol (**1b**, $C_{12}H_{10}O_2$, newly synthesized, M.W. 186.0681). The bracketed value is the calculated *m/z* value. RSD = experimental *m/z* value ÷ calculated *m/z* value.

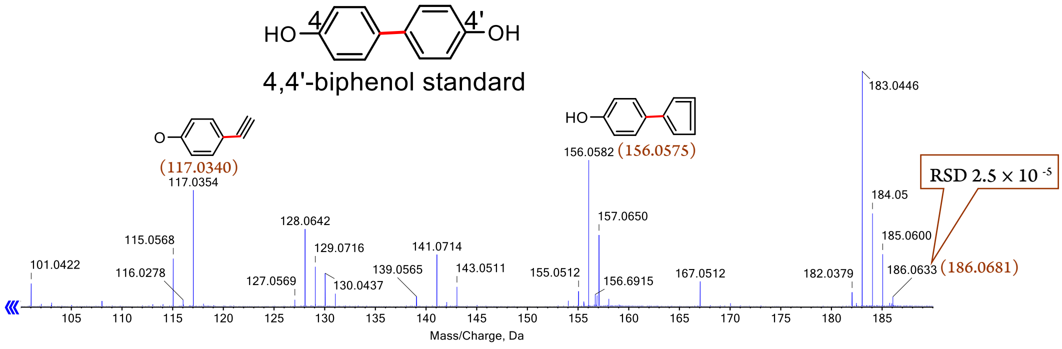

Fig. S1.3 The molecular ion peak and MS/MS spectra of standard 4,4'-biphenol (**1c**, $C_{12}H_{10}O_2$, CAS 92-88-6, M.W. 186.0681) The bracketed value is the calculated *m/z* value. RSD = experimental *m/z* value ÷ calculated *m/z* value.

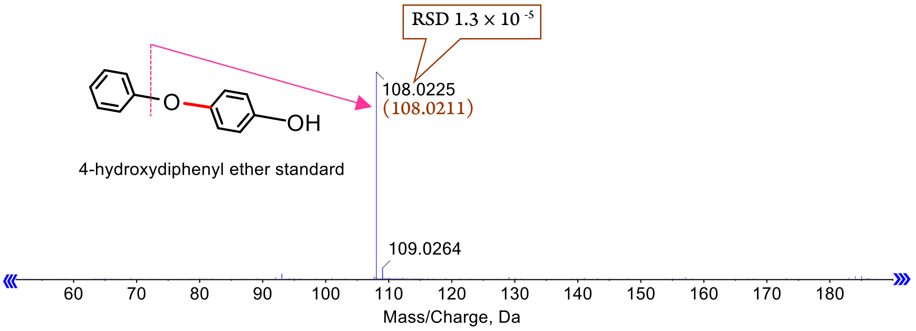

Fig. S1.4 The molecular ion peak and MS/MS spectra of standard 4-hydroxyldiphenyl ether (**1d**, $C_{12}H_{10}O_2$, CAS 831-82-3, M.W. 186.0681)

The bracketed value is the calculated *m/z* value. RSD = experimental *m/z* value ÷ calculated *m/z* value.

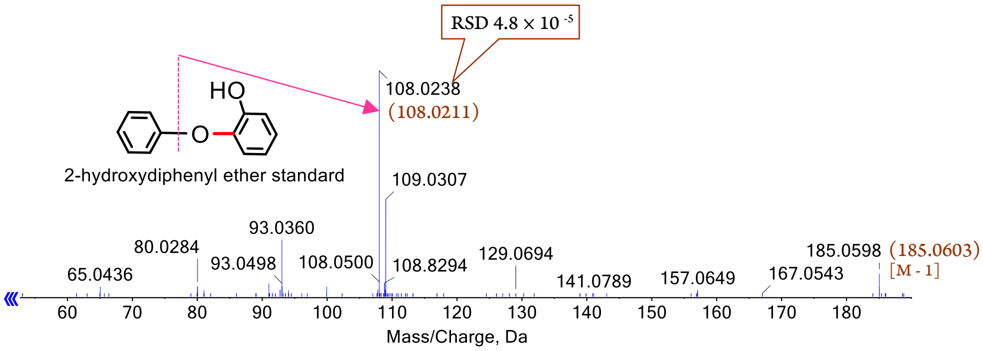

Fig. S1.5 The molecular ion peak and MS/MS spectra of standard 2-hydroxyldiphenyl ether (**1e**, $C_{12}H_{10}O_2$, CAS 2417-10-9, M.W. 186.0681).

The bracketed value is the calculated *m/z* value. RSD = experimental *m/z* value ÷ calculated *m/z* value.

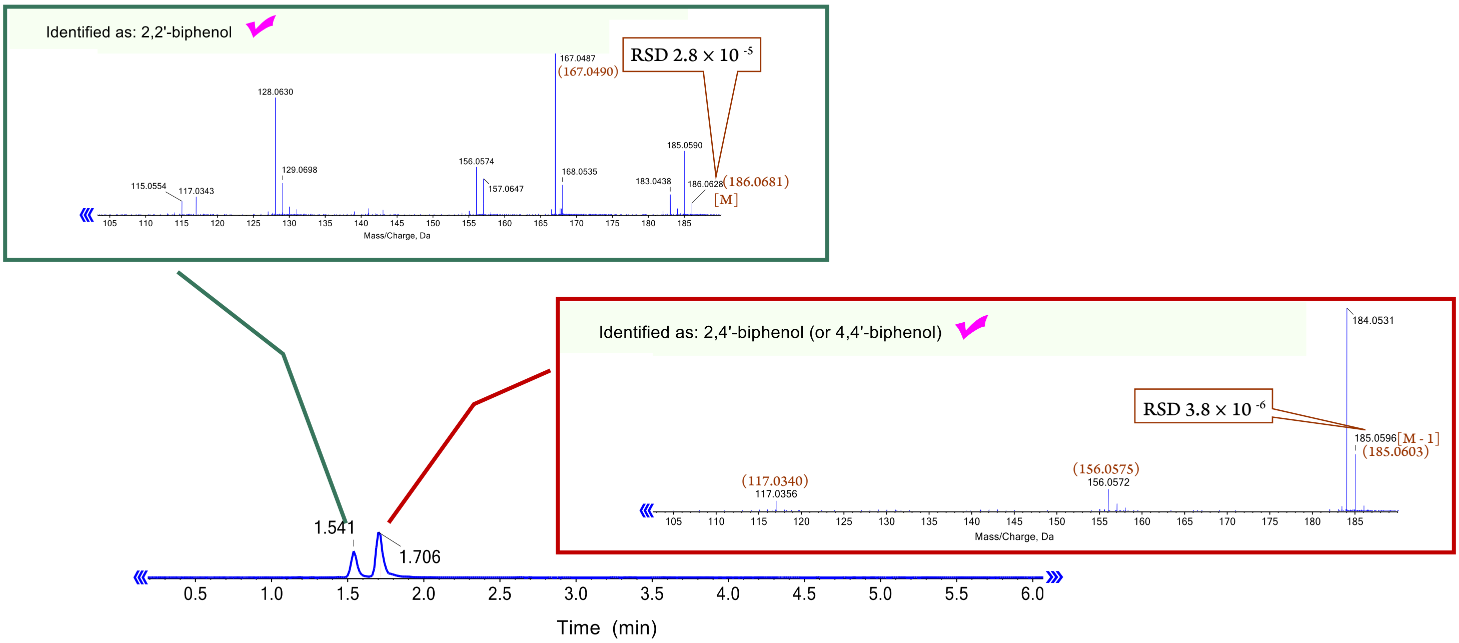

Fig. S1.6 The main UHPLC–ESI-Q-TOF-MS results of dimerization products of DPPH$^{\bullet}$-treated phenol (**1**) in methanol and product identifications.
The chromatographic peaks were extracted using formula [$C_{12}H_{10}O_2$ – H]. The bracketed value is the calculated *m/z* value. RSD = experimental *m/z* value ÷ calculated *m/z* value. The product peak was identified through comparison of the molecular ion peak and MS/MS data with the standards.

The 1.541 min peak was fully identified as 2,2’-biphenol (**1a**). The identification was based on the evidence that, (1) molecular ion peak showed very low RSD value (10^{-5}) comparison with the calculated M.W.; (2) The MS spectra profile of product was similar to that of standard 2,2’-biphenol; (3) the characteristic fragment *m/z* 167, a result of adjacent site elimination effect (i.e., - H_2O). [3]

The 1.706 min peak was identified as 2,4’-biphenol **1b** (or 4,4’-biphenol **1c**). The identification was based on two characteristic fragments *m/z* 117 and 156. Although **1b** and **1c** were not strictly recognized, however, it cannot hinder the discussion regarding the C–C bonding domination rule and meta-excluded rule in the main text.

The exclusion of two ethers (4-hydroxyldiphenyl ether **1d** and 2-hydroxyldiphenyl ether **1e**) was based on *m/z* 108, the characteristic fragment and basic peak, which was elucidated in **Fig. S1.4-S1.5**.

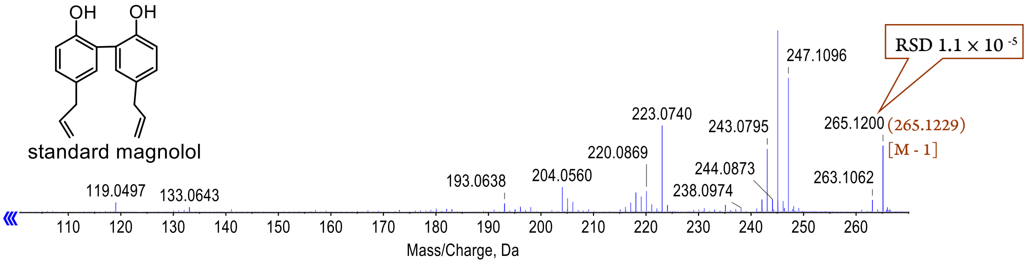

Fig. S2.1 The molecular ion peak and MS/MS spectra of standard magnolol (**2a**, $C_{18}H_{18}O_2$, CAS number: 528-43-8, M.W. 266.1307). The bracketed value is the calculated *m/z* value. RSD = experimental *m/z* value ÷ calculated *m/z* value.

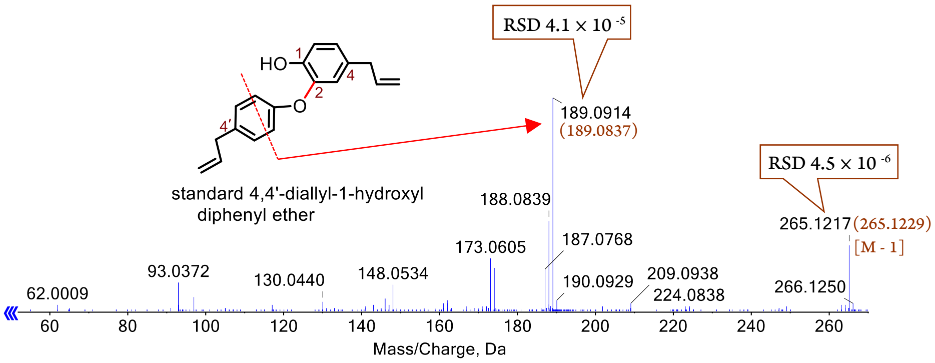

Fig. S2.2 The molecular ion peak and MS/MS spectra of standard 4,4'-diallyl-1-hydroxyldiphenyl ether (**2b**, $C_{18}H_{18}O_2$, newly synthesized, M.W. 266.1307).
The bracketed value is the calculated *m/z* value. RSD = experimental *m/z* value ÷ calculated *m/z* value.

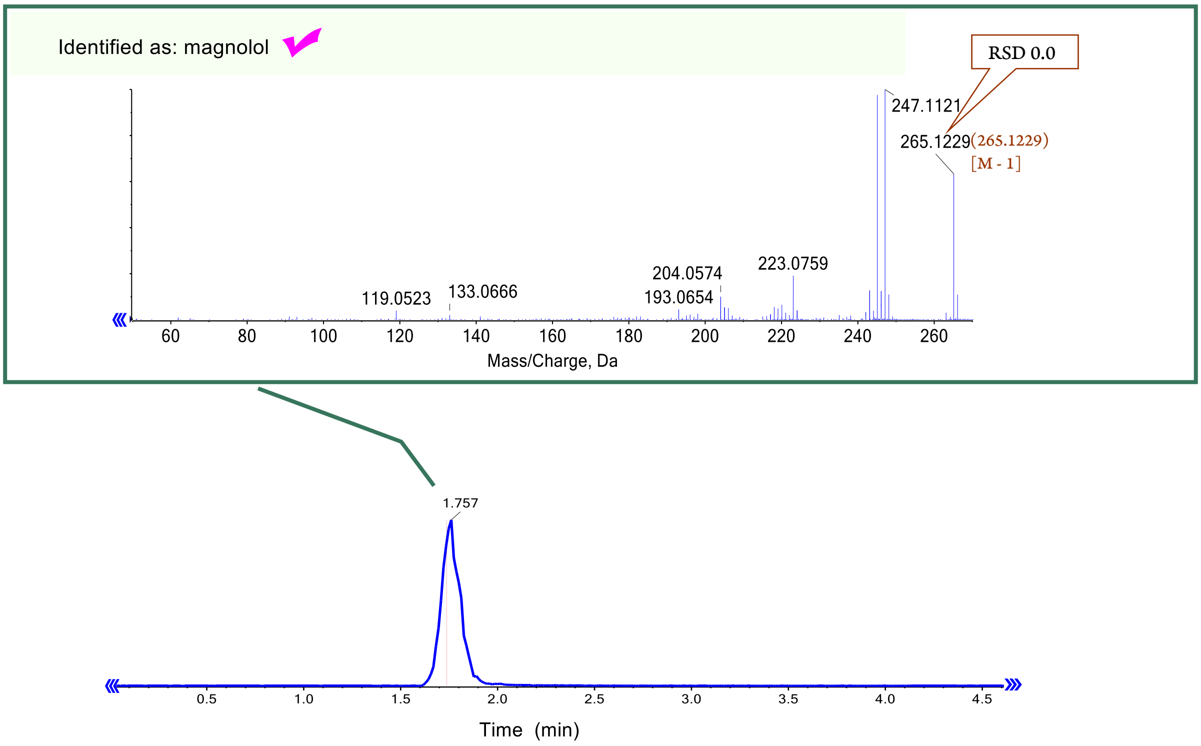

Fig. S2.3 The main UHPLC–ESI-Q-TOF-MS results of dimerization products of DPPH•-treated 4-allylphenol (**2**) in methanol and product identifications. The chromatographic peaks were extracted using formula [$C_{18}H_{18}O_2$ – H]. The bracketed value is the calculated *m/z* value. RSD = experimental *m/z* value ÷ calculated *m/z* value.

The product peak was identified through comparison of the molecular ion peak and MS/MS data with the standards. The 1.757 min peak was thus identified as magnolol (**2a**); and 4,4'-diallyl-1-hydroxyldiphenyl ether (**2b**) was excluded for the absence of characteristic fragment *m/z* 189 in the product MS/MS spectra.

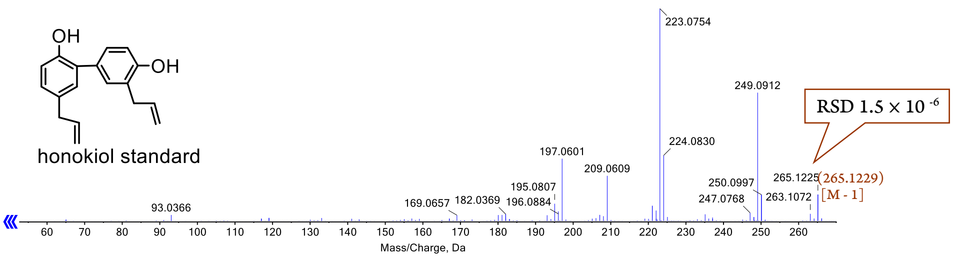

Fig. S3.1 The molecular ion peak and MS/MS spectra of standard honokiol (**3a**, $C_{18}H_{18}O_2$, CAS number: 35354-74-6, M.W. 266.1307). The bracketed value is the calculated *m/z* value. RSD = experimental *m/z* value ÷ calculated *m/z* value.

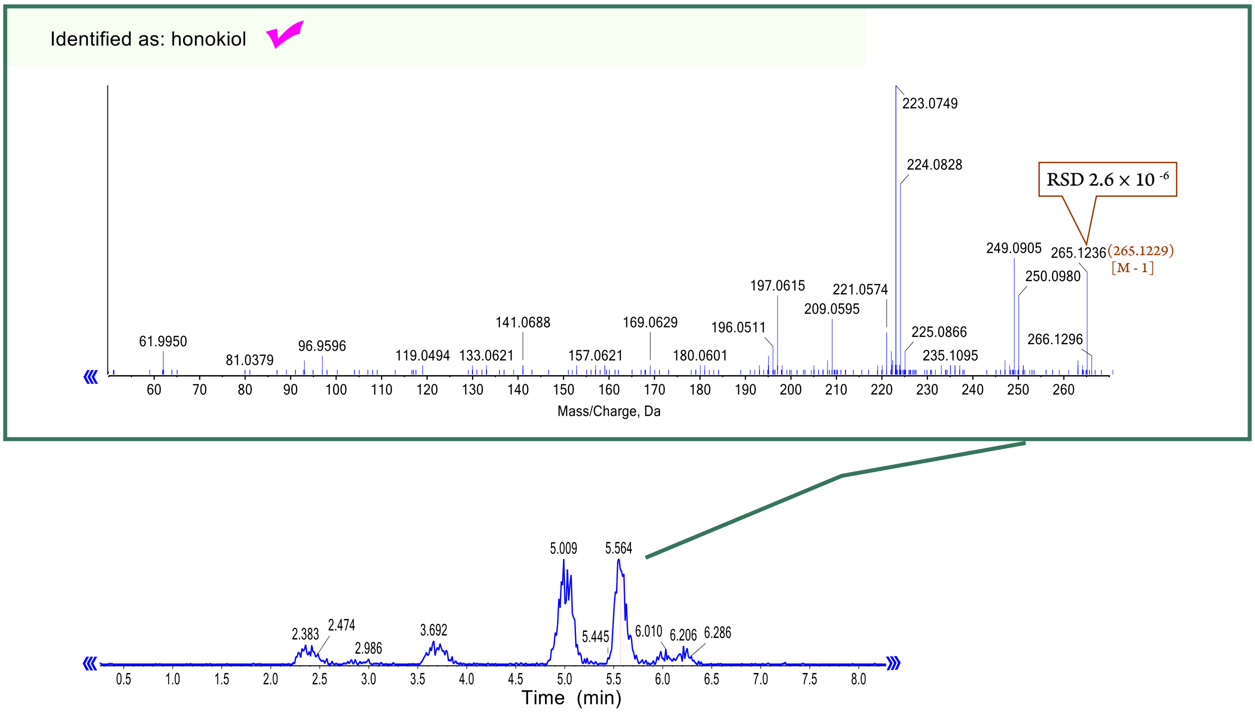

Fig. S3.2 The main UHPLC–ESI-Q-TOF-MS results of products of DPPH•-treated 4-allylphenol (**2**) *plus* 2-allyphenold (**3**) in methanol and product identification.

The chromatographic peaks were extracted using formula [$C_{18}H_{18}O_2$ – H]. The bracketed value is the calculated *m/z* value. RSD = experimental *m/z* value ÷ calculated *m/z* value. The product peak was identified through comparison of the molecular ion peak and MS/MS data with the standards. The 5.564 min peak was thus identified as honokiol (**3a**).

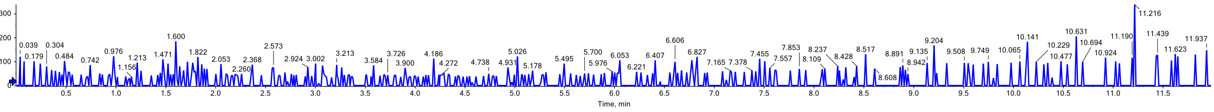

Fig. S4.1 The chromatographic peaks of dimerization products of DPPH•-treated syringic acid (**4**) in methanol. (The peaks extracted using formula [$C_{18}H_{18}O_{10}$ – H])

There was no dimeric product.

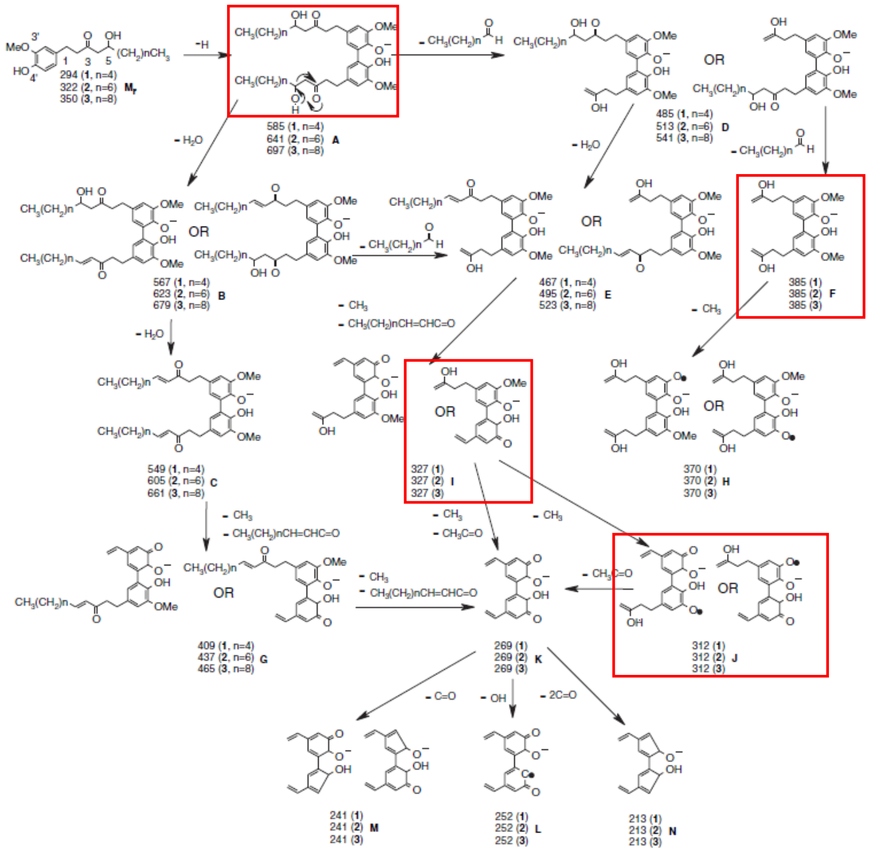

Fig. S5.1 The screenshot of documented data and elucidation of [6]-gingerol 6,6'-dimer (**5a**) and [10]-gingerol 6,6'-dimer (**6a**) [4].

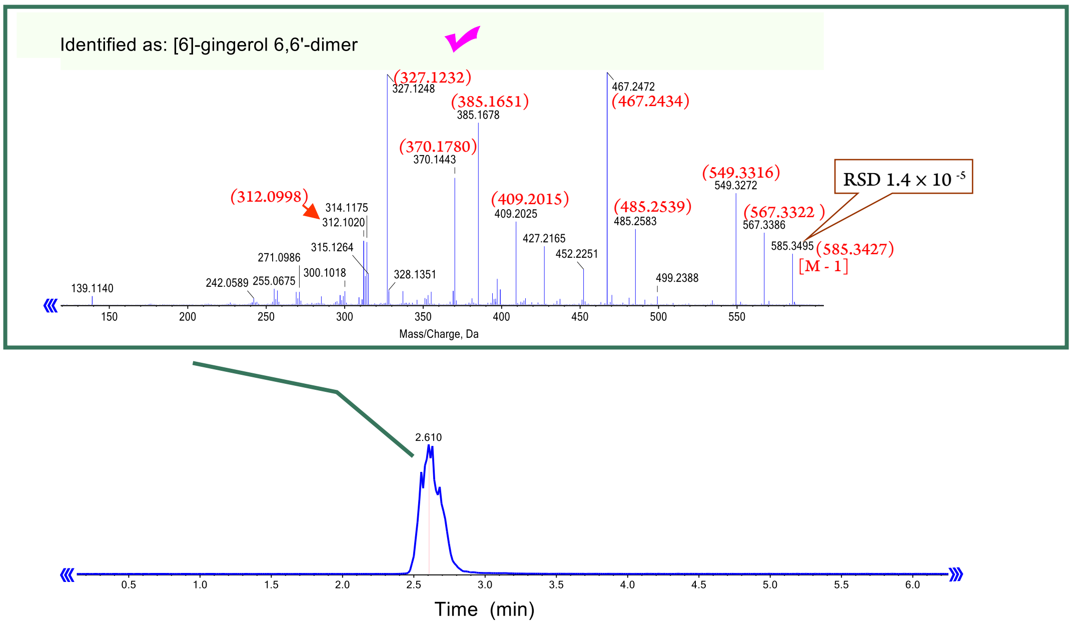

Fig. S5.2 The main UHPLC–ESI-Q-TOF-MS results of dimerization products of DPPH•-treated [6]-gingerol (**5**) in methanol and [6]-gingerol 6,6' dimer (**5a**) identification.

The chromatographic peaks were extracted using formula [$C_{34}H_{50}O_8$ – H]. The bracketed value in red is the calculated *m/z* value. RSD = experimental *m/z* value ÷ calculated *m/z* value. The product peak was identified through comparison of the documented molecular ion peak and MS/MS data[4]. The 2.610 min peak was thus identified as [6]-gingerol 6,6' dimer (**5a**).

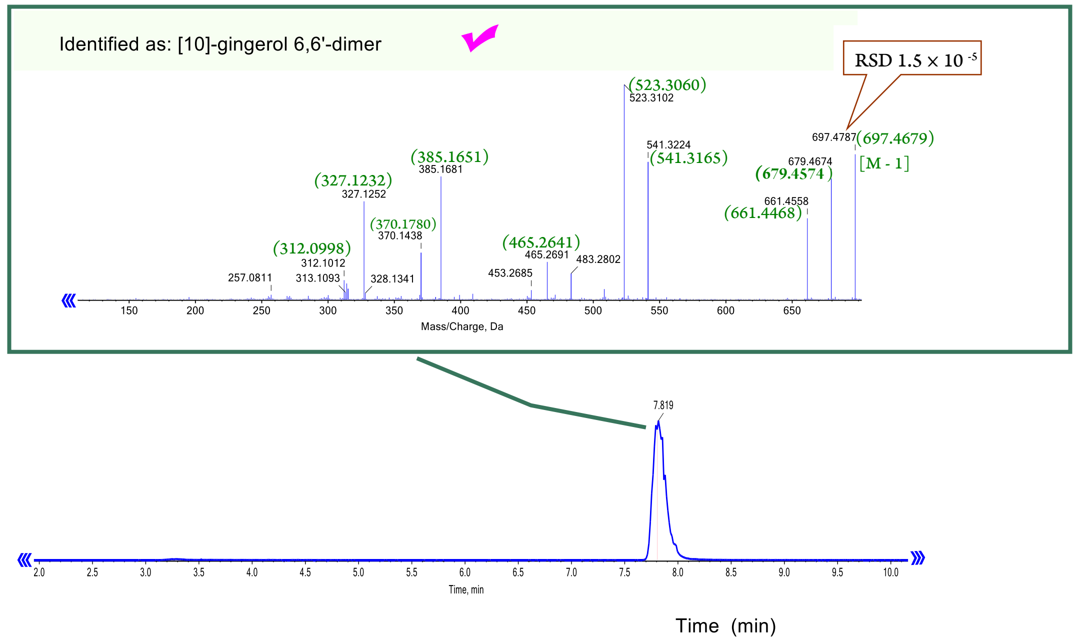

Fig. S6.1 The main UHPLC–ESI-Q-TOF-MS results of dimerization products of DPPH•-treated [10]-gingerol (**6**) in methanol and product identification.

The chromatographic peaks were extracted using formula [$C_{42}H_{66}O_8$ – H]. The bracketed value in green is the calculated *m/z* value. RSD = experimental *m/z* value ÷ calculated *m/z* value. The product peak was identified through comparison of the documented molecular ion peak and MS/MS data (green in **Fig. S5.1**) [4]. The 7.819 min peak was thus identified as [10]-gingerol 6,6’ dimer (**6a**).

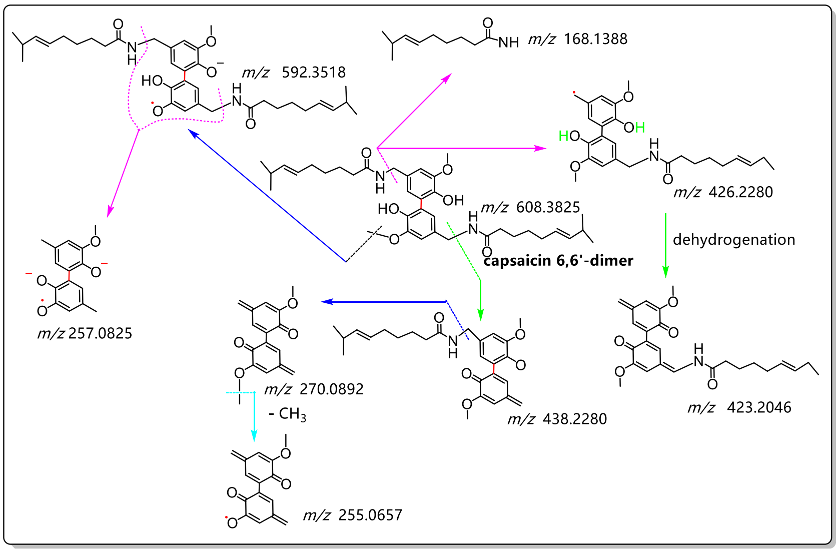

Fig. S7.1 The MS elucidation of capsaicin 6,6'-dimer (**7a**).
The structure, molecular ion peak, and some fragmenting pathways of capsaicin 6,6'-dimer (**7a**) were proposed by the previous study [5].

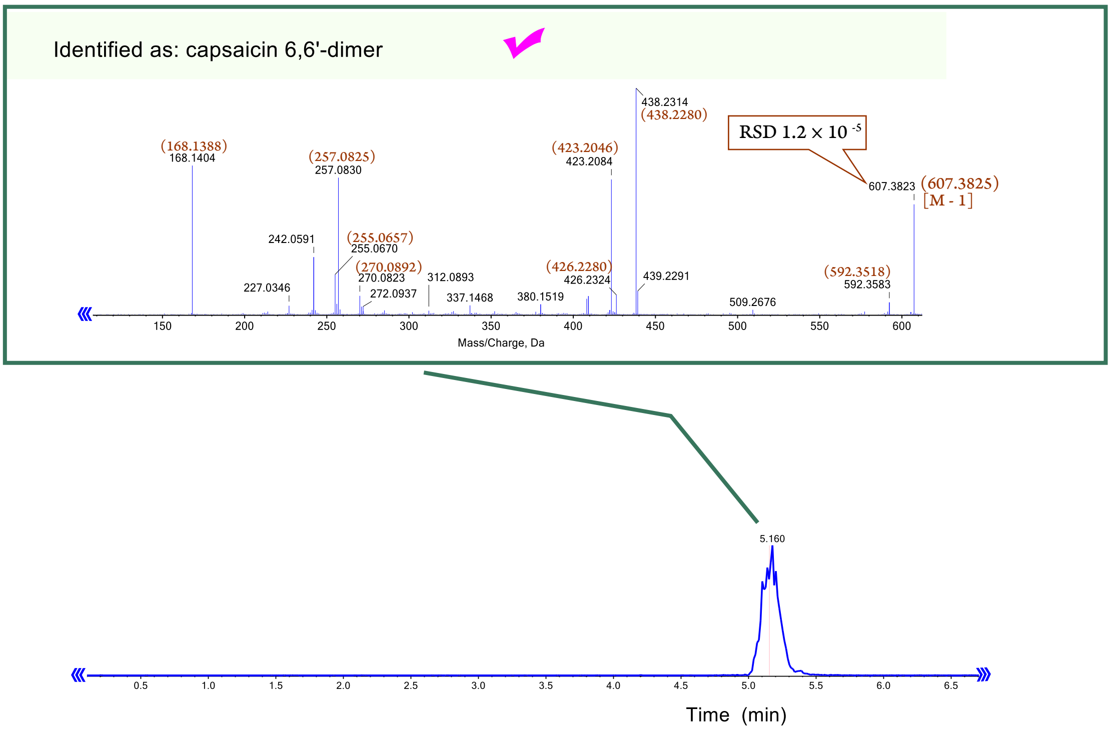

Fig. S7.2 The main UHPLC–ESI-Q-TOF-MS results of dimerization products of DPPH•-treated capsaicin (**7**) in methanol and product identification.
The chromatographic peaks were extracted using formula [$C_{36}H_{52}N_2O_6$ – H]. The bracketed value is the calculated *m/z* value, which was cited from **Fig. S7.1**. RSD = experimental *m/z* value ÷ calculated *m/z* value. The product peak was identified through comparison of the documented molecular ion peak and MS/MS data (**Fig. S7.1**) [5]. The 5.160 min peak was thus identified as capsaicin 6,6'-dimer (**7a**).

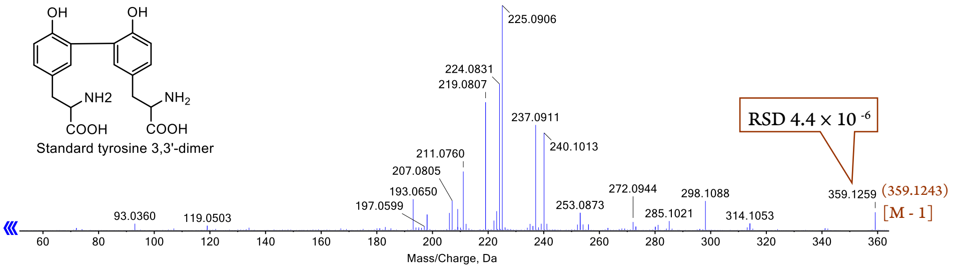

Fig. S8.1 The molecular ion peak and MS/MS spectra of standard tyrosine 3,3'-dimer (**8a**, $C_{18}H_{20}N_2O_6$, newly synthesized, M.W. 360.1321).

The bracketed value is the calculated *m/z* value. RSD = experimental *m/z* value ÷ calculated *m/z* value.

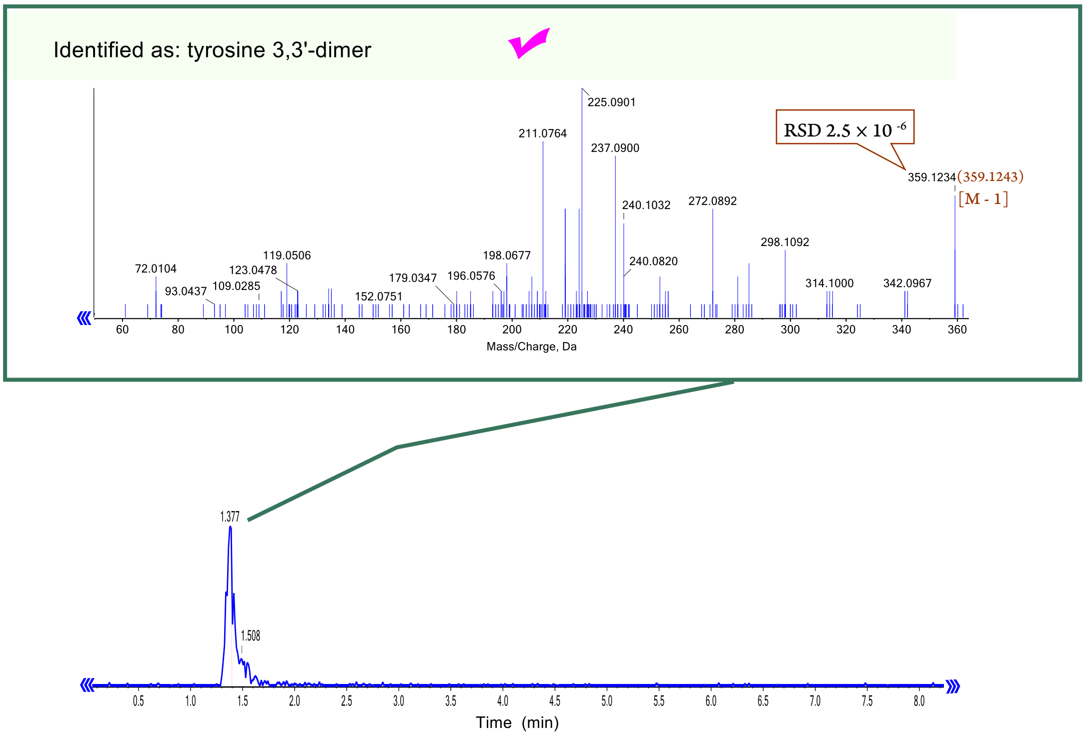

Fig. S8.2 The main UHPLC–ESI-Q-TOF-MS results of dimerization products of DPPH$^{\bullet}$-treated tyrosine (**8**) in methanol and product identification.

The chromatographic peaks were extracted using formula [$C_{18}H_{20}N_2O_6$ – H]. The bracketed value is the calculated *m/z* value. RSD = experimental *m/z* value ÷ calculated *m/z* value. The product peak was identified through comparison of the molecular ion peak and MS/MS data of standard tyrosine 3,3'-dimer (**8a**). The 1.377 min peak was thus identified as tyrosine 3,3'-dimer (**8a**).

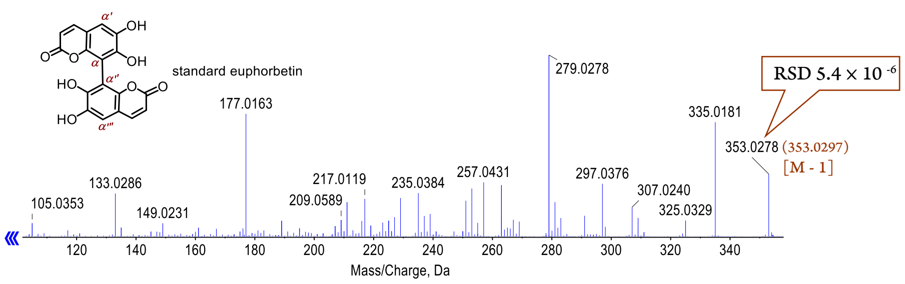

Fig. S9.1 The molecular ion peak and MS/MS spectra of standard euphorbetin (**9a**, $C_{18}H_{10}O_8$, CAS 35897-99-5, M.W. 354.0376). The bracketed value is the calculated *m/z* value. RSD = experimental *m/z* value ÷ calculated *m/z* value.

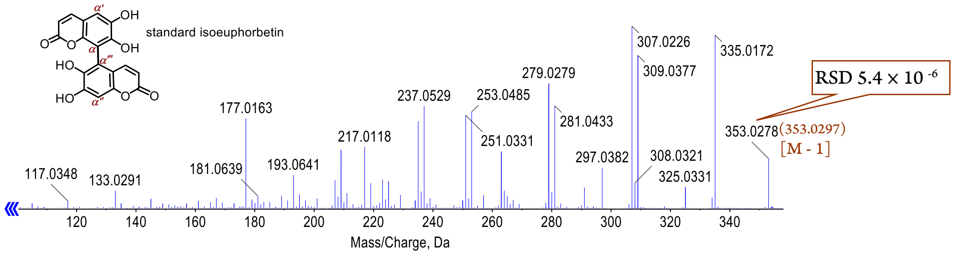

Fig. S9.2 The molecular ion peak and MS/MS spectra of standard isoeuphorbetin (**9b**, $C_{18}H_{10}O_8$, CAS 50677-55-9, M.W. 354.0376). The bracketed value is the calculated *m/z* value. RSD = experimental *m/z* value ÷ calculated *m/z* value.

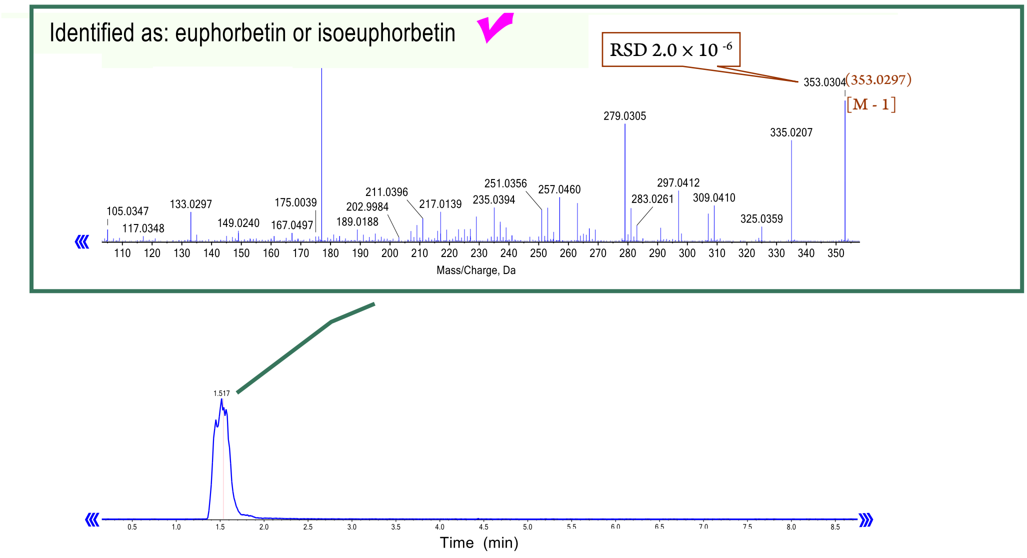

Fig. S9.3 The main UHPLC–ESI-Q-TOF-MS results of dimerization products of DPPH•-treated esculetin (9) in methanol and product identification.

The chromatographic peaks were extracted using formula [$C_{18}H_{10}O_8$ – H]. The bracketed value is the calculated *m/z* value. RSD = experimental *m/z* value ÷ calculated *m/z* value. The product peak was identified through comparison of the molecular ion peak and MS/MS data of standard euphorbetin (**9a**) and isoeuphorbetin (**9b**).

The 1.517 min peak was thus identified as **9a** or **9b**. Although the two isomers were not strictly distinguished, however, it cannot hinder the discussion regarding the basic rules in the main text.

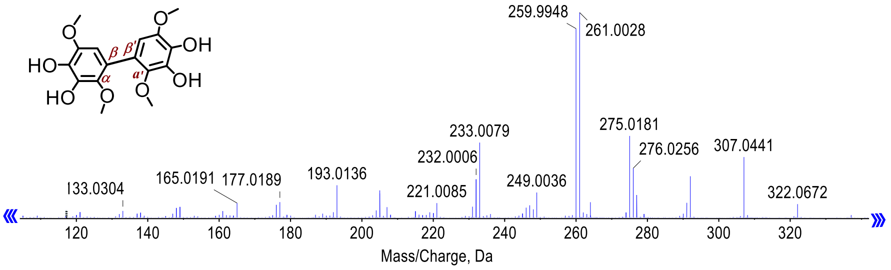

Fig. S10.1 The molecular ion peak and MS/MS spectra of standard 3,6-dimethoxycatechol β,β'-dimer (**10**, newly synthesized, $C_{16}H_{18}O_8$, M.W. 338.1002).
The bracketed value is the calculated *m/z* value. RSD = experimental *m/z* value ÷ calculated *m/z* value.

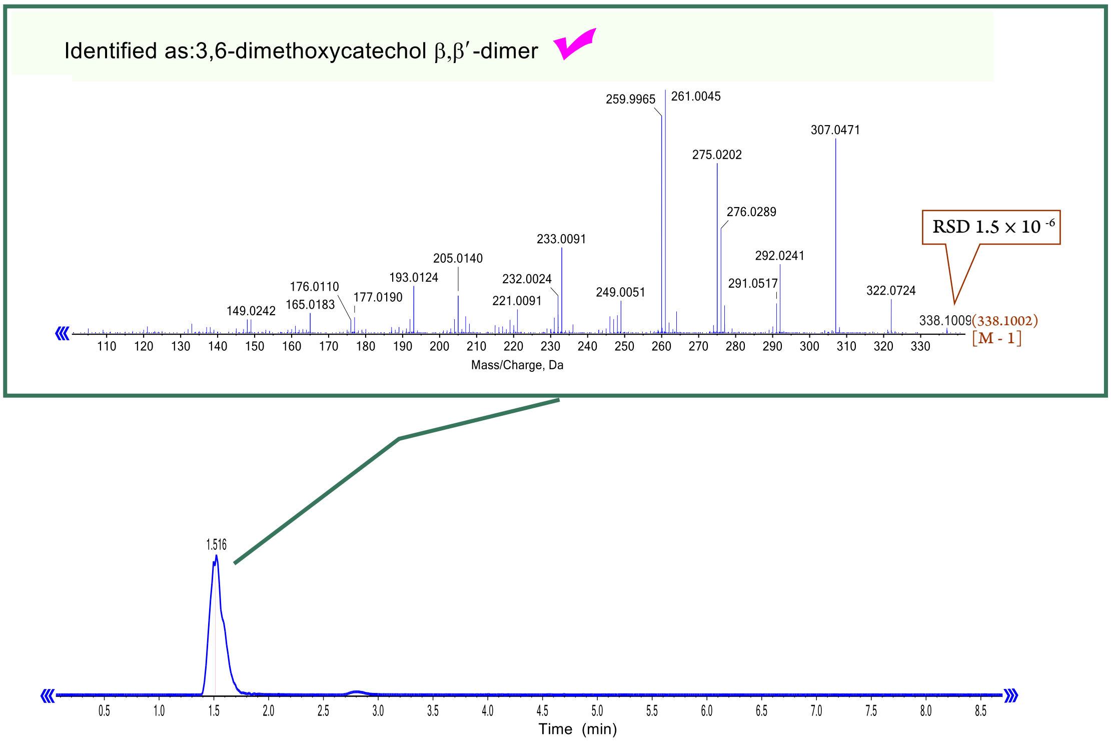

Fig. S10.2 The main UHPLC–ESI-Q-TOF-MS results of dimerization products of DPPH•-treated 3,6-dimethoxycatechol (**10**, newly synthesized) in methanol and product identification.
The chromatographic peaks were extracted using formula [$C_{16}H_{18}O_8$ – H]. The bracketed value is the calculated *m/z* value. RSD = experimental *m/z* value ÷ calculated *m/z* value. The product peak was identified through comparison of the molecular ion peak and MS/MS data of standard 3,6-dimethoxycatechol β,β'-dimer (**10a**). The 1.516 min peak was thus identified as 3,6-dimethoxycatechol β,β'-dimer (**10a**).

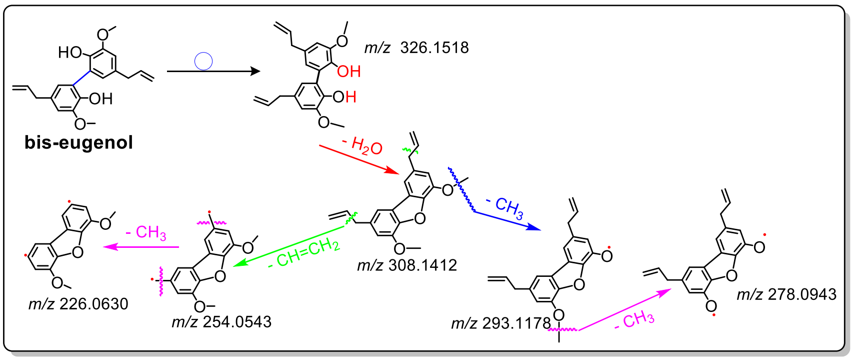

Fig. S11.1 The MS elucidation of bis-eugenol (**11a**, i.e., dehydrodieugenol, eugenol 6.6'-dimer, M.W. 326.1518).
The structure, molecular ion peak, and some fragmenting pathways of bis-eugenol (**11a**) were proposed by the previous study [6]. The circle "◯" indicates the rotation of σ-bond. The dehydration (- H_2O) in red is a consequence of adjacent elimination effect. [3]

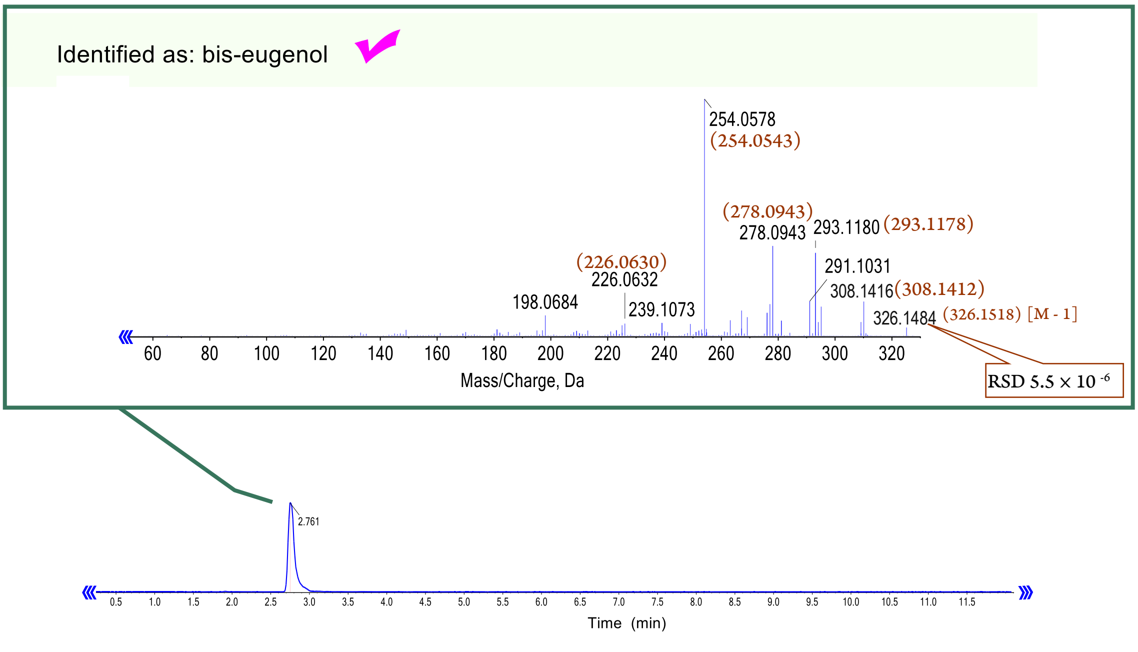

Fig. S11.2 The main UHPLC–ESI-Q-TOF-MS results of dimerization products of DPPH•-treated eugenol (**11**) in methanol and product identification.
The chromatographic peaks were extracted using formula [$C_{20}H_{22}O_4$ – H]. The bracketed value is the calculated *m/z* value, which was cited from **Fig. S11.1**. RSD = experimental *m/z* value ÷ calculated *m/z* value. The product peak was identified through comparison of the documented molecular ion peak and MS/MS data (**Fig. S11.1**) [6]. The 2.761 min peak was thus identified as bis-eugenol (**11a**). The characteristic and basic peak (*m/z* 254.0578) can be used to distinguish from dehydrodiisoeugenol (**12a**).

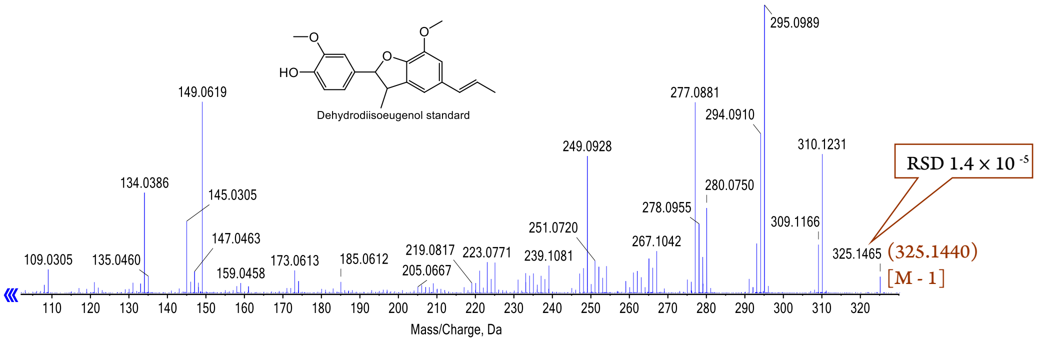

Fig. S12.1 The molecular ion peak and MS/MS spectra of standard dehydrodiisoeugenol (**12a**, CAS 2680-81-1, $C_{20}H_{22}O_4$, M.W. 326.1518).
The bracketed value is the calculated m/z value. RSD = experimental m/z value ÷ calculated m/z value.

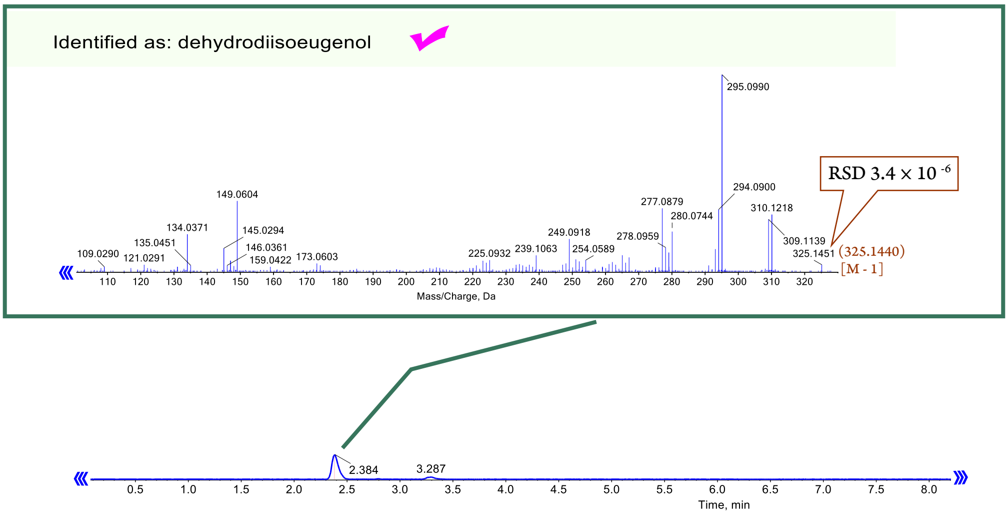

Fig. S12.2 The main UHPLC–ESI-Q-TOF-MS results of dimerization products of DPPH•-treated isoeugenol (**12**) in methanol and product identification.
The chromatographic peaks were extracted using formula [$C_{20}H_{22}O_4$ – H]. The bracketed value is the calculated m/z value. RSD = experimental m/z value ÷ calculated m/z value. The product peak was identified through comparison of the molecular ion peak and MS/MS data of standard dehydrodiisoeugenol (**12a**). The 2.384 min peak was thus identified as dehydrodiisoeugenol (**12a**).

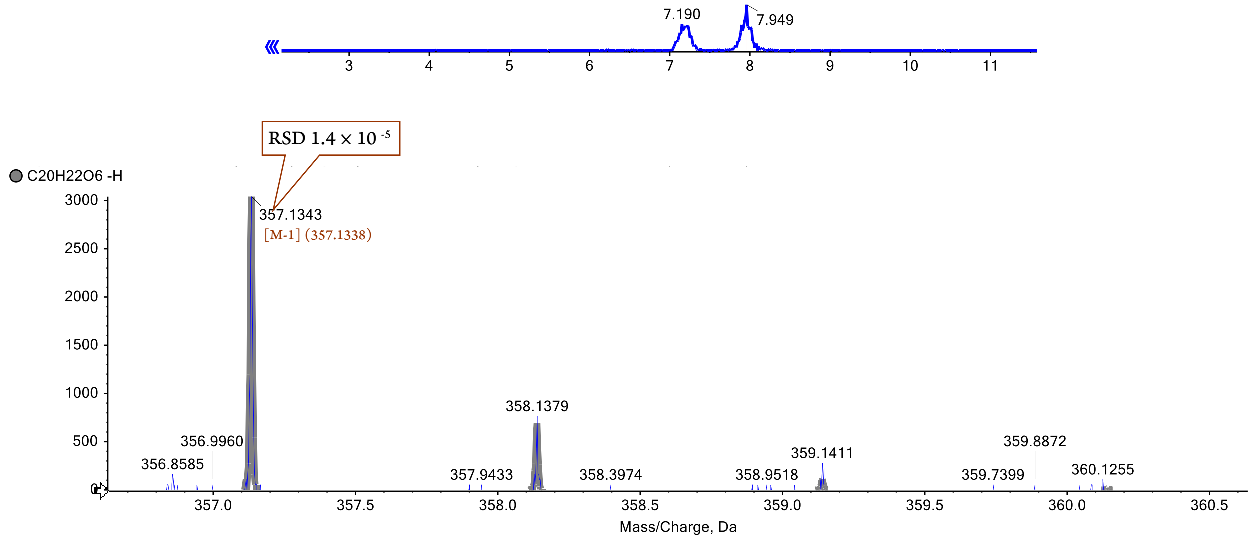

Fig. S13.1 The typical UHPLC–ESI-Q-TOF-MS results of dimerization products of DPPH$^{\bullet}$-treated coniferyl alcohol (**13**) in methanol. The chromatographic peaks were extracted using formula [$C_{20}H_{22}O_6$ – H]. The bracketed value is the calculated *m/z* value. RSD = experimental *m/z* value ÷ calculated *m/z* value.

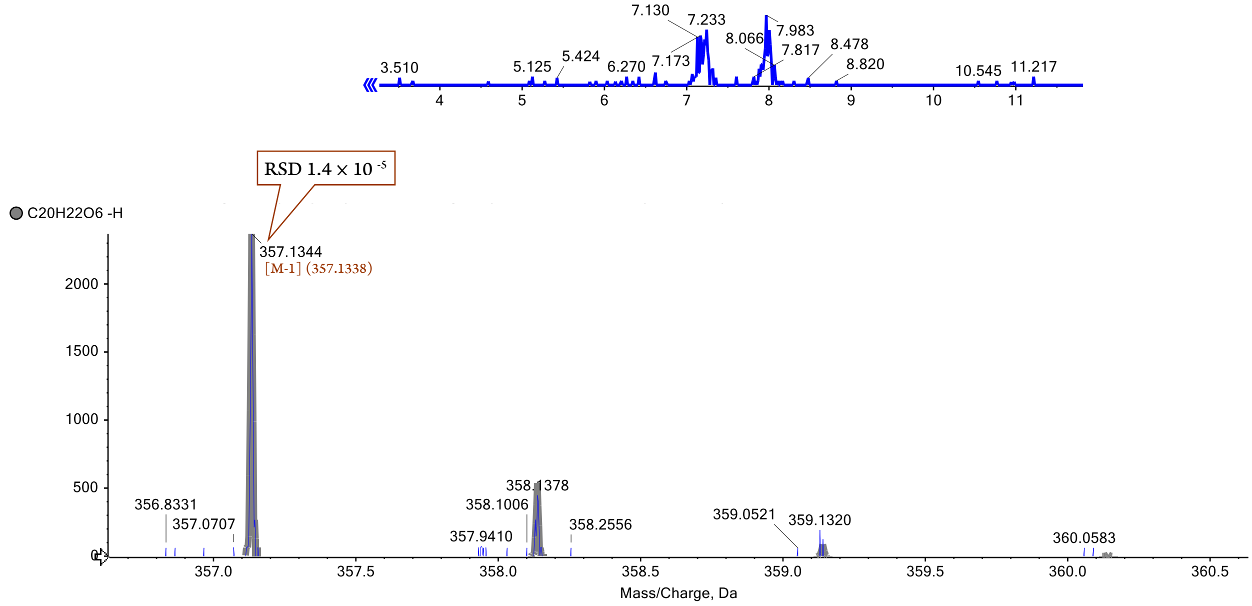

Fig. S13.2 The typical UHPLC–ESI-Q-TOF-MS results of dimerization products of HRP-catalyzed coniferyl alcohol (**13**). The chromatographic peaks were extracted using formula [$C_{20}H_{22}O_6$ – H]. The bracketed value is the calculated *m/z* value. RSD = experimental *m/z* value ÷ calculated *m/z* value.

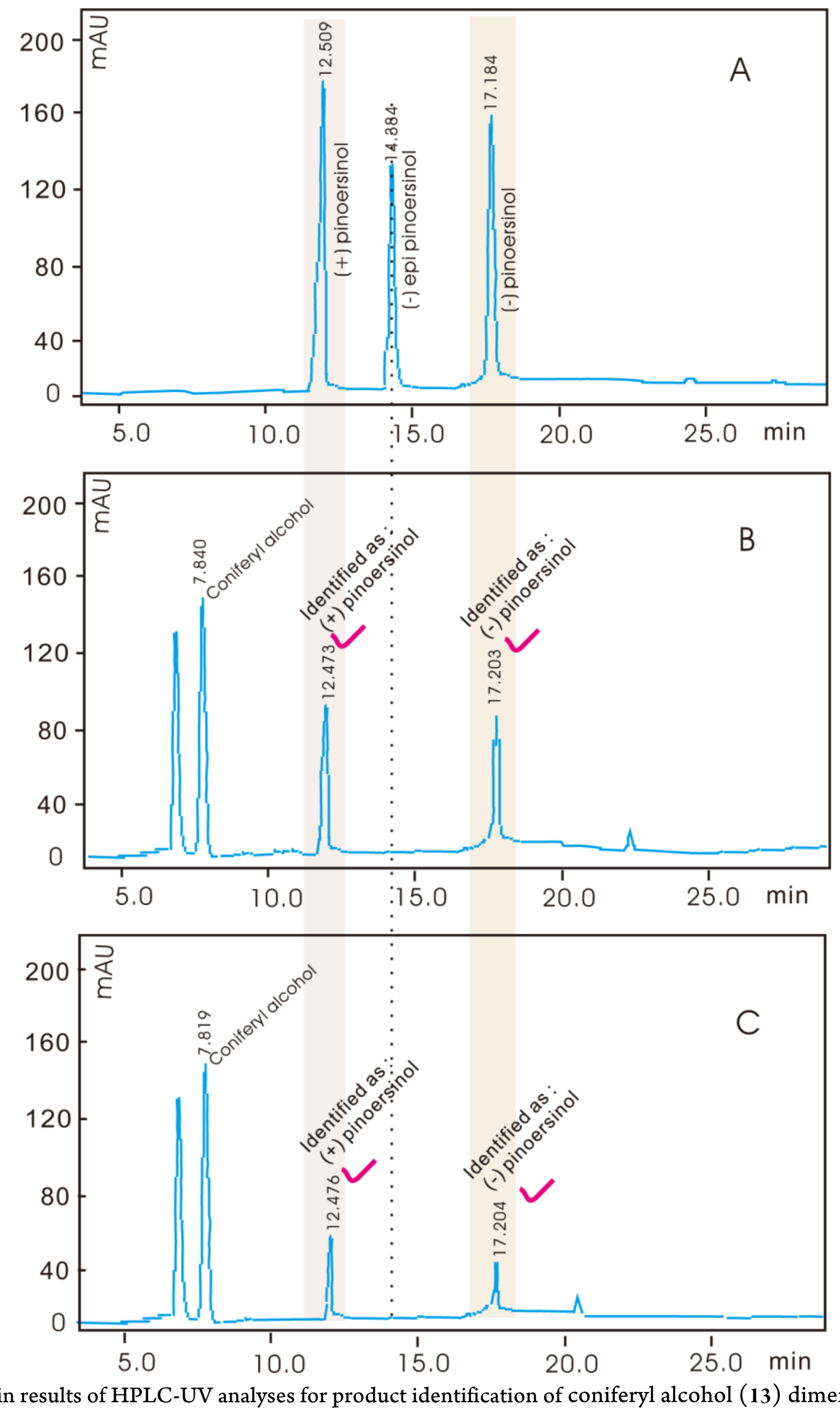

Fig. S13.3 he main results of HPLC-UV analyses for product identification of coniferyl alcohol (**13**) dimerization: A, spectra of standard (+) pinoresinol [(+) (**13a**)], (-) pinoresinol [(-) (**13a**)], and (-) epipinoresinol [(-) (**13b**)]; B, dimerization products of DPPH$^{\bullet}$-treated coniferyl alcohol (**13**) in methanol; C, dimerization products of HRP-catalyzed coniferyl alcohol (**13**).

Agilent 1260 (Agilent Technologies Inc., Palo Alto, CA, USA) equipped with **Chiral**-MD(2)-RH Phenomenex (250 mm × 4.6 mm, 5 μm) (Torrance, CA, USA). The mobile phase: 0.1% H_3PO_4 : ACN (50:50); Injection volume: 5 μL; flow rate: 0.5 mL/min; Determination absorDPPHn wavelength 280 nm.

Product identification and exclusion: To preliminarily idetify the dimeric products, the products were determined using in UHPLC–ESI-Q-TOF-MS anaysis *in situ*. As seen in Fig. S13.1 and Fig. S13.2, DPPH$^{\bullet}$-treated and HRP-catalyzed coniferyl alcohol (**13**) respectively

produced two chromatographic peaks with [$C_{20}H_{22}O_6$ – H] formula and the M.W. $C_{20}H_{22}O_6$. The documents suggested these peaks as pinoresinols [7]. Consulting with the method [7], these peaks were further distinguished using chiral column-based HPLC-UV analysis. As seen in **Fig. S13.3**, the dimeric products of coniferyl alcohol (**13**) were proven to be (+) pinoresinol [(+) (**13a**)] and (-) pinoresinol [(-) (**13a**)]. However, there was no (-) epipinoresinol [(-) (**13b**)].

References

[1] Tahiliani, M.; Koh, K.P.; Shen, Y.; Pastor, W.A.; Bandukwala, H.; Brudno, Y.; Agarwal, S.; Iyer, L.M.; Liu, D.R.; Aravind, L.; Rao, A. Conversion of 5-methylcytosine to 5-hydroxymethylcytosine in mammalian DNA by MLL partner TET1. *Science*. **2009**, 324, 930-935.

[2] He, Y.F.; Li, B.Z.; Li, Z.; Liu, P.; Wang, Y.; Tang, Q.; Ding, J.; Jia, Y.; Chen, Z.; Li, L.; Sun, Y.; Li, X.; Dai, Q.; Song, C.X.; Zhang, K.; He, C.; Xu, G.L. Tet-mediated formation of 5-carboxylcytosine and its excision by TDG in mammalian DNA. *Science*. **2011**, 333, 1303-1307.

[3] J.H., G., *Mass spectrometry*. 2013, Beijing: Science press.

[4] Jiang, H.; Somogyi, A.; Timmermann, B.N.; Gang, D.R. Instrument dependence of electrospray ionization and tandem mass spectrometric fragmentation of the gingerols. *Rapid Commun Mass Spectrom*. **2006**, 20, 3089-3100.

[5] Tian, K.; Zhu, J.; Li, M.; Qiu, X. Capsaicin is efficiently transformed by multiple cytochrome P450s from Capsicum fruit-feeding Helicoverpa armigera. *Pestic Biochem Physiol*. **2019**, 156, 145-151.

[6] Dos Santos Grecco, S.; Letsyo, E.; Tempone, A.G.; Ghilardi Lago, J.H.; Jerz, G. Electrospray mass-spectrometry guided target isolation of neolignans from Nectandra leucantha (Lauraceae) by high performance- and spiral-coil countercurrent chromatography. *J Chromatogr A*. **2019**, 1608, 460422.

[7] Davin, L.B.; Wang, H.B.; Crowell, A.L.; Bedgar, D.L.; Martin, D.M.; Sarkanen, S.; Lewis, N.G. Stereoselective bimolecular phenoxy radical coupling by an auxiliary (dirigent) protein without an active center. *Science*. **1997**, 275, 362-366.
