# Supplementary material for: Phytophenol Dimerization Reaction: From Basic Rules to Diastereoselectivity and Beyond
Source: Molecules. 2022 Jul 28;27(15):4842. doi: 10.3390/molecules27154842 (PMC9369853; doi:10.3390/molecules27154842)
Supplement: Supplementary file 1 [file molecules-27-04842-s001.zip › Supplementary File S2 Experimental & Computational Details.pdf]

Suppl. 2- Experimental & Computational Details

Phytophenol Dimerization Reaction: From Basic Rules to Diastereoselectivity and Beyond

Shuqin Liu [1,†], Xican Li [1,*,†], Ban Chen [1], Xiaojian Ouyang [1], Yulu Xie [1] and Dongfeng Chen [2,*]

1 School of Chinese Herbal Medicine, Guangzhou University of Chinese Medicine, Guangzhou Higher Education Mega Center, Waihuang East Road No. 232, Guangzhou 510006, China; 20201110616@stu.gzucm.edu.cn (S.L.); imchenban@foxmail.com(B.C.); oyxiaojian55@163.com (X.O.); xieyulu1900@163.com (Y.X.)

2 School of Basic Medical Science, Guangzhou University of Chinese Medicine, Guangzhou Higher Education Mega Center, Waihuang East Road No. 232, Guangzhou 510006, China

* Correspondence: lixican@126.com (X.L.); chen888@gzucm.edu.cn (D.C.)

† These authors contributed equally to this work.

Experimental

Chemicals

Table S2.1 All chemicals used in the study

No	Name	CAS	Formula	Purity	Supplier
**1**	phenol	108-95-2	C_6H_6O	99%	Nuoke Technology Development Co., Ltd. (Tianjin, China)
**1a**	2,2'-biphenol	1806-29-7	$C_{12}H_{10}O_2$	97%	J&K Scientific Ltd. (Beijing, China)
**1b**	2,4'-biphenol		$C_{12}H_{10}O_2$		Newly synthesized
**1c**	4,4'-biphenol	92-88-6	$C_{12}H_{10}O_2$	97%	J&K Scientific Ltd. (Beijing, China)
**1d**	4-hydroxyldiphenyl ether (4-phenoxyphenol)	831-82-3	$C_{12}H_{10}O_2$	97%	J&K Scientific Ltd. (Beijing, China)
**1e**	2-hydroxyldiphenyl ether (2-phenoxyphenol)	2417-10-9	$C_{12}H_{10}O_2$	97%	J&K Scientific Ltd. (Beijing, China)
**2**	syringic acid	530-57-4	$C_9H_{10}O_5$	98%	BioBioPha Co., Ltd. (Kunming, China)
**3**	4-allylphenol	501-92-8	$C_9H_{10}O$	95%	Shaoyuan Reagent Co., Ltd. (Shanghai, China)
**3a**	magnolol	528-43-8	$C_{18}H_{18}O_2$	98%	Alfa Biotechnology Co., Ltd. (Chengdu, China)
**3b**	4,4'-diallyl-1-hydroxyldiphenyl ether	No available	$C_{18}H_{18}O_2$	98%	Shenzhen Xinyao Biotechnology Co., Ltd. (Shenzhen, China)
**4**	2-allylphenol	1745-81-9	$C_9H_{10}O$	95%	J&K Scientific Ltd. (Beijing, China)
**4a**	honokiol	35354-74-6	$C_{18}H_{18}O_2$	98%	Alfa Biotechnology Co., Ltd. (Chengdu, China)
**5**	[6]-gingerol	23513-14-6	$C_{17}H_{26}O_4$	98%	Biopurify Phytochemicals Co., Ltd. (Chengdu, China)
**6**	[10]-gingerol	23513-15-7	$C_{21}H_{34}O_4$	98%	Alfa Biotechnology Co., Ltd. (Chengdu, China)
**7**	capsaicin	404-86-4	$C_{18}H_{27}NO_3$	99%	J&K Scientific Ltd. (Beijing, China)
**8**	tyrosine	556-03-6	$C_9H_{11}NO_3$	99%	J&K Scientific Ltd. (Beijing, China)
**8 a**	3,3'-dityrosine		$C_{18}H_{20}O_6$		Newly synthesized
**9**	esculetin	305-01-1	$C_9H_6O_4$	98%	Alfa Biotechnology Co., Ltd. (Chengdu, China)
**9a**	isoeuphorbetin	50677-55-9	$C_{18}H_{10}O_8$	98%	ChemFaces Co., Ltd. (Wuhan, China)
**9b**	euphorbetin	35897-99-5	$C_{18}H_{10}O_8$	98%	Alfa Biotechnology Co., Ltd. (Chengdu, China).
**10**	3,6-dimethoxylcatechol	No available	$C_8H_{10}O_4$	98%	Shenzhen Xinyao Biotechnology Co., Ltd. (Shenzhen, China)
**10a**	3,6-dimeothylcatechol *β,β'*-dimer	No available	$C_{16}H_{18}O_8$	98%	Shenzhen Xinyao Biotechnology Co., Ltd. (Shenzhen, China)
**11**	eugenol	97-53-0	$C_{10}H_{12}O_2$	98%	Alfa Biotechnology Co., Ltd. (Chengdu, China)
**12**	isoeugenol	97-54-1	$C_{10}H_{12}O_2$	99%	Sigma-Aldrich Shanghai Trading Co., Ltd. (Shanghai, China).
**12a**	dehydrodiisoeugenol	2680-81-1	$C_{20}H_{22}O_4$	98%	Alfa Biotechnology Co., Ltd. (Chengdu, China)
**13**	coniferyl alcohol	32811-40-8	$C_{10}H_{12}O_3$	98%	Alfa Biotechnology Co., Ltd. (Chengdu, China)
(+) **13a**	(+) pinoresinol	487-36-5	$C_{20}H_{22}O_6$	98%	BioBioPha Co., Ltd. (Kunming, China)
(-) **13a**	(-) pinoresinol	81446-29-9	$C_{20}H_{22}O_6$	98%	BioBioPha Co., Ltd. (Kunming, China)
**13b**	(-) epipinoresinol	10061-38-8	$C_{20}H_{22}O_6$	98%	BioBioPha Co., Ltd. (Kunming, China)
---	Phenylboronic acid	98-80-6	$C_6H_5B(OH)_2$	A.R.	Sigma-Aldrich Shanghai Trading Co., Ltd. (Shanghai, China)
---	4-iodophenol	540-38-5	C_6H_5IO	A.R.	Sigma-Aldrich Shanghai Trading Co., Ltd. (Shanghai, China)
---	3-methoxycatechol	934-00-9	$C_7H_8O_3$	A.R.	Sigma-Aldrich Shanghai Trading Co., Ltd. (Shanghai, China)
---	Horseradish peroxidase	9003-99-0	lyophilized powder, ~150 U/mg		Sigma-Aldrich Shanghai Trading Co., Ltd. (Shanghai, China)
---	DPPH radical	1898-66-4	$C_{18}H_{12}N_5O_6^{\bullet}$		Sigma-Aldrich Shanghai Trading Co., Ltd. (Shanghai, China)
---	glacial acetic acid	64-19-7	$C_2H_4O_2$	A.R.	J&K Scientific (Beijing, China)
---	Water	7732-18-5	H_2O		HPLC grade, Merck KGaA (Darmstadt, Germany)
---	Formic acid	64-18-6	HCOOH		HPLC grade, Merck KGaA (Darmstadt, Germany)
---	Methanol	67-56-1	CH_3OH		HPLC grade, Merck KGaA (Darmstadt, Germany)

Note: The chemicals include the phytophenol monomers, identified dimeric products, three excluded dimeric products, and synthetic materials.

*Synthesis of 2,4'-biphenol (2,4'-dihydroxybiphenyl, **1b**)*

The synthesis of 2,4'-biphenol (**1b**) was based on the Suzuki-Miyaura coupling reaction [1], and performed under the argon gas protection [2]. One equiv. 4-iodophenol and appropriate boronic acid were mixed and suspended in water. To the suspension, four equiv. K_2CO_3 was added, followed by Pd/C. The mixture was heated for 3 h at 75 °C. Then, it was cooled into room temperature, followed by acidification and ether extraction. The ether layer was separated and further dried using $MgSO_4$. The ether solvent was removed by nitrogen-blow. The residue was purified by silica column chromatography using *n*-hexane/diethyl ether mixtures as gradient eluents. A white solid was obtained (Fig. S1). **ESI-MS** (**ESI**) calcd for $C_{12}H_{10}O_2$ [M^+]: 186.0837, found: 186.0641. **^{1}H-NMR** and **^{13}C-NMR** spectra (Fig. S2-3) were consistent with the literature data of 2,4'-biphenol (**1b**) [3].

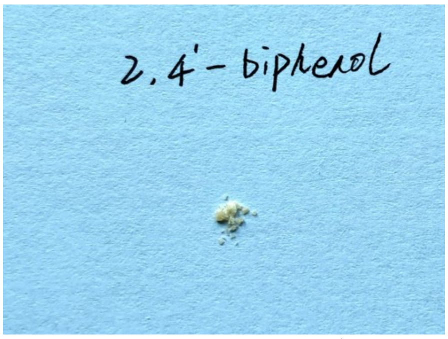

Fig. S1 Photo of 2,4'-biphenol (2,4'-dihydroxybiphenyl, **1b**)

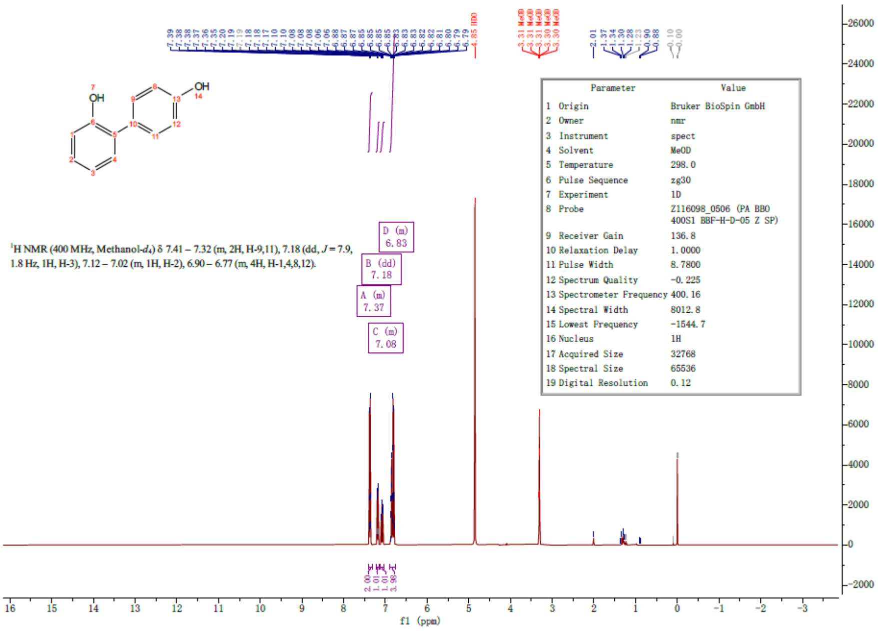

Fig. S2 ^{1}H-NMR of 2,4'-biphenol (2,4'-dihydroxybiphenyl, **1b**)

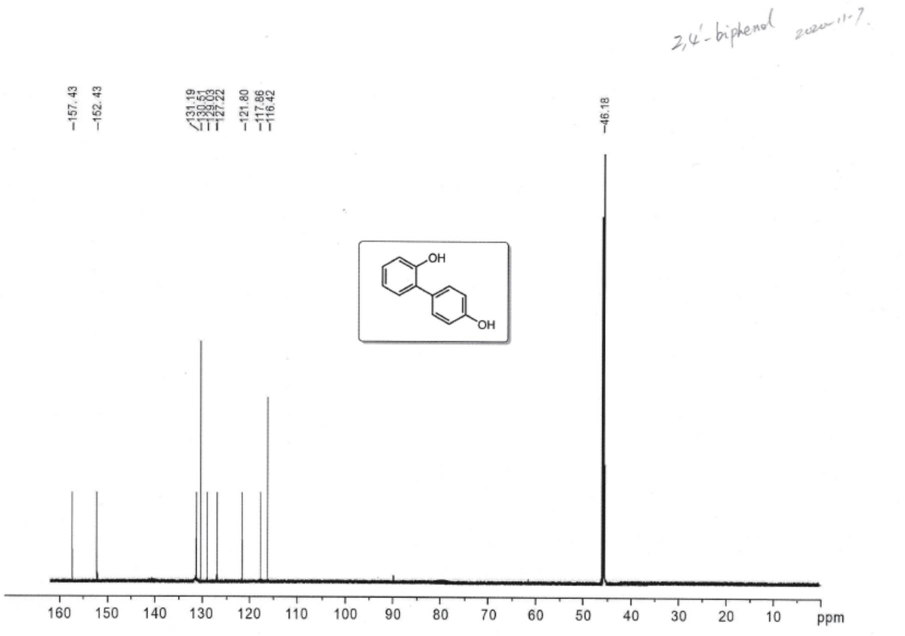

Fig. S3 ^{13}C-NMR of 2,4'-biphenol (2,4'-dihydroxybiphenyl, **1b**, solvent methanol, 100 MHz)

*Synthesis of 3,3'-dityrosine (**8a**)*

The preparation of 3,3'-dityrosine (**8a**) was based on a simple and convenient method [4]. In brief, 5 mM tyrosine solution was prepared in 0.2 M phosphate (pH 2.1). Oxidation was initiated by adding 5 Mm Mn (III). After 1 min reaction at 25 °C, the mixture was filtered to remove any undissolved Mn (III) acetate and the filtrate was dried under vacuum at 80 °C. Ammonia water was added in excess to remove the phosphate by precipitation. After filtration, the remaining ammonia water was evaporated under vacuum at 80 °C. Using the difference in the solubility of tyrosine and dityrosine at low pH and low temperature, unreacted tyrosine remaining in the dry material was mostly eliminated by washing with cold acidic water (4 °C, pH 2) and filtration. The filtration was recrystallized to yield a colorless powder (Fig. S6). ESI-MS (EI) calcd for $C_{18}H_{20}N_2O_6$ [M^+]: 360.1321, found: 360.1306. ^{1}H-NMR and ^{13}C-NMR spectra (Fig. S7-8) were consistent with the literature data of 3,3'-dityrosine (**8a**) [4].

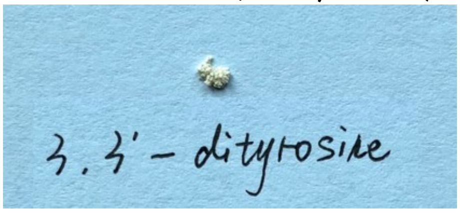

Fig. S4 Photo of 3,3'-dityrosine (**8a**)

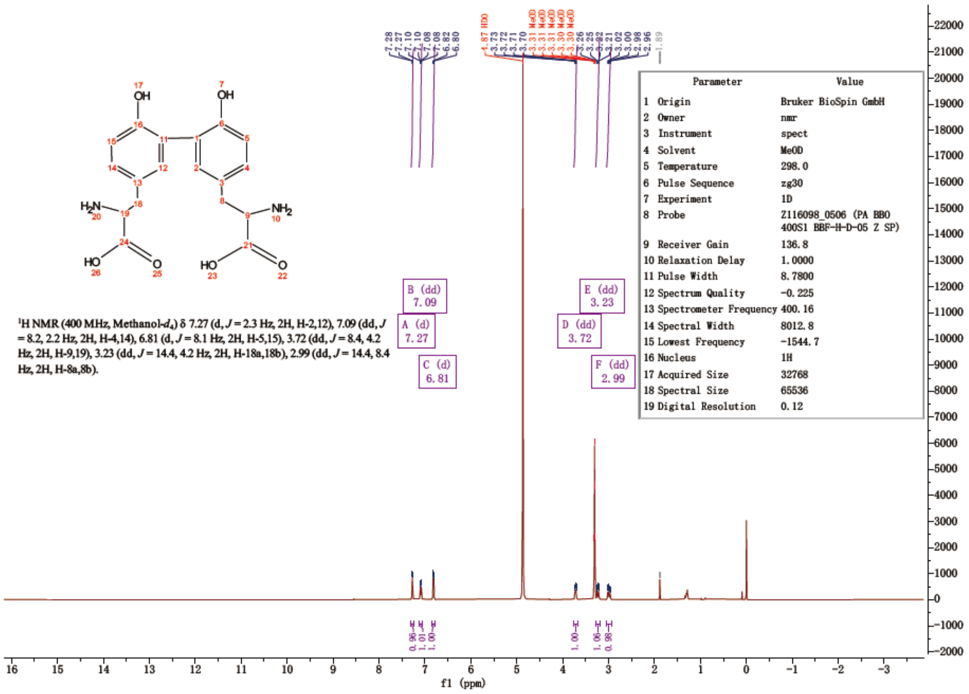

Fig. S5 ^{1}H-NMR spectra of 3,3'-dityrosine (**8a**)

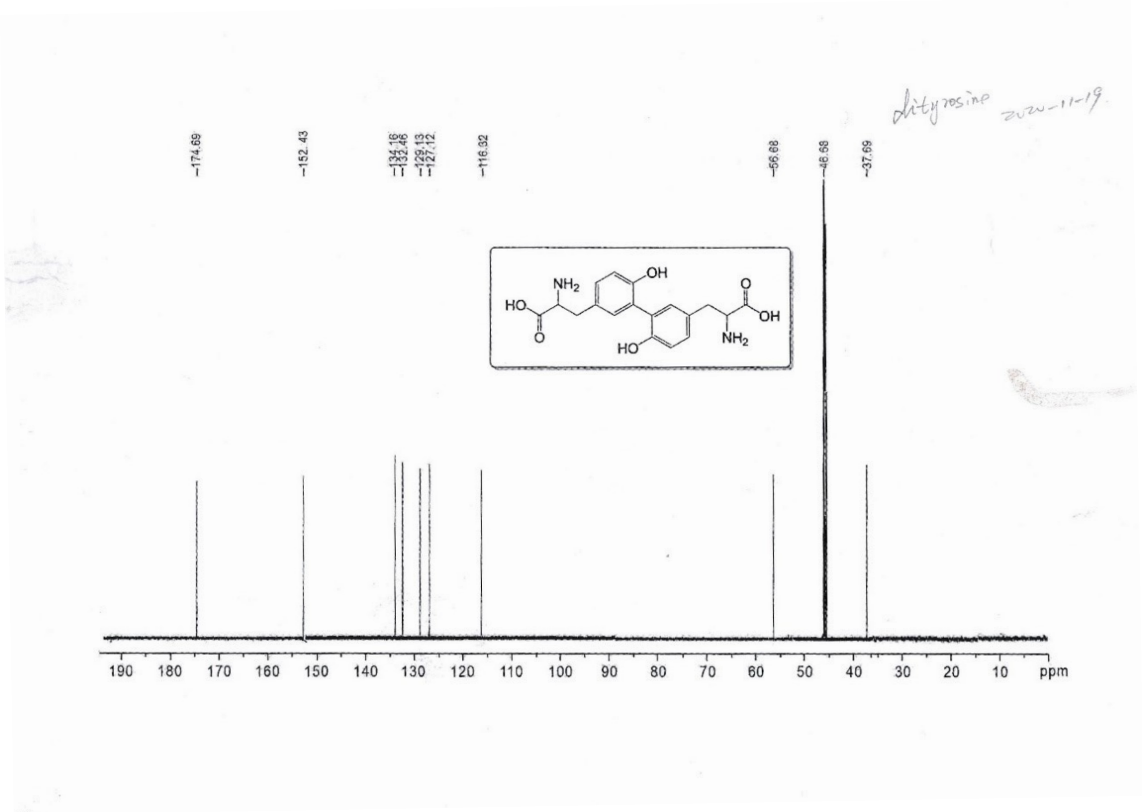

Fig. S6 ^{13}C-NMR spectra of 3,3'-dityrosine (**8a**, solvent methanol, 100 MHz)

Radical coupling reaction experiments of phytophenols

The DPPH$^•$-initiated PRC reaction was conducted as the following. The DPPH radical work solution was prepared at 16 μM in methanol. The phenol (**1**) sample solution was mixed with DPPH radical work solution at 4:1 molar ratio. The mixture was incubated in the dark at room temperature. The above experiments were repeated using 4-allylphenol (**2**), syringic acid (**4**), [6]-gingerol (**5**), [8]-gingerol (**6**), capsaicin (**7**), tyrosine (**8**), esculetin (**9**), 3,6-dimethoxylcatechol (**10**), eugenol (**11**), isoeugenol (**12**), and coniferyl alcohol (**13**). The experiments were also repeated using 4-allylphenol (**2**) *plus* 2-allylphenol (**3**). After 12 hours, the reaction was terminated by addition of 40-time volume methanol. The methanol solution was passed through a 0.22 μm filter. The filtrate was stored under 4℃ for further analysis.

The horseradish peroxidase (HRP)-catalyzed PRC reaction of coniferyl alcohol was conducted as the following. 8.3 U/mL HRP was prepared using K_2HPO_4/KH_2PO_4 buffer (pH6.0), and then was mixed with coniferyl alcohol methanol solution (120 μL, 5.55 mM). To initiate the reaction, 34 μL H_2O_2 (30%) was added to the mixture. The total volume of reaction mixture was adjusted to 1000 μL using the above buffer. The 1000 μL reaction system was incubated at 20℃. After 60 min, the product mixture was extracted using ethyl acetate for twice. The extract was centrifugated at 5000 r/min for 10 min for twice. The supernatants were collected and then dried into residue. The residue was re-resolved using 1.0 mL methanol. The methanol solution was passed through a 0.22 μm filter. The filtrate was stored under 4℃ for UPLC-ESI-Q-TOF-MS analysis and HPLC-UV analyses.

UPLC-ESI-Q-TOF-MS analysis for PRC products and standards

The filtrates were performed using UHPLC-ESI-Q-TOF-MS analysis. The UHPLC-ESI-Q-TOF-MS analysis apparatus was equipped with a Chiral-MD(2)-RH Phenomenex column (2.1 mm i.d. ×100 mm, 1.6 μm, Phenomenex Inc., Torrance, CA, USA). The mobile phase in the chromatography was made up of a mixture of methanol (phase A) and 0.1% formic acid in water (phase B). The column was eluted at a flow rate of 0.3 mL/min with the following gradient elution program: 0–2 min, 30% B; 2–10 min, 30% → 0% B; 10–12 min, 0% → 30% B. The sample injection volume was 3 μL. The Q-TOF-MS analysis was conducted on a Triple TOF 5600*plus* mass spectrometer (AB SCIEX, Framingham, MA, USA) equipped with an ESI source, which was run in the negative and positive ionization mode. The scan range was set at 50–1600 Da. The system was run with the following parameters: ion spray voltage, −4500 V; ion source heater, 550 °C; curtain gas (CUR, N2), 30 psi; nebulizing gas (GS1, air), 50 psi; and Turbo Ion Spray (TIS) gas (GS2, air), 50 psi. The declustering potential (DP) was set at −100 V, and the collision energy (CE) was set at −45 V with a collision energy spread (CES) of 15 V.

The newly synthesized and authentic standards were dissolved in methanol below 100 μg/mL and then passed through a 0.22 μm filter. The filtrate was analyzed using UHPLC-ESI-Q-TOF-MS under the same determination condition.

HPLC-UV analysis for PRC products of coniferyl alcohol

To preliminarily identify the possible PRC products, the product mixture of DPPH•-treated coniferyl alcohol was analyzed using HPLC-UV analysis. In brief, the above product mixture was analyzed by HPLC on Agilent 1260 (Agilent Technologies Inc., Palo Alto, CA, USA), which was equipped with Chiral-MD(2)-RH Phenomenex (250 mm × 4.6 mm, 5 μm) (Torrance, CA, USA). The mobile phase consisted of 0.1% H_3PO_4 and ACN (50:50). The injection volume and flow rate were 5 μL and 0.5 mL/min; The column temperature and determination absorption wavelength were 35 °C and 280 nm respectively. The retention time of (+) pinoresinol, (-) pinoresinol, and (-) epipinoresinol were compared with the authentics in the assays.

The above protocol was repeated using the product mixture of HRP/H_2O_2-treated coniferyl alcohol, to preliminarily identify the possible PRC products,

Molecular weight calculation

The molecular weight calculation based on the formula is vital for comparison with the m/z values from the Q-TOF-MS analysis. In the present study, the molecular weight calculations were conducted based on the accurate relative atomic masses. The relative atomic masses of C, H, O, and N were 12.0000, 1.007825, 15.994915, and 14.003074, respectively [5].

Computational details

To ensure correlations between chemical experiments, all calculations were performed in the methanol solvation model based on density (SMD) using the Gaussian 16 software [6]. The geometry optimization and vibration frequency of all molecules were calculated at the B3LYP-D3(BJ)/6–311+G** level, while the single-point energies were calculated using M06-2X-D3 hybrid functional combined with a larger basis set def2-TZVPD based on optimized geometries with no imaginary frequency [7]. The calculation formula for the BDE, DPE and ΔG is as follow:

$$\mathrm{BDE} = H\,(\mathrm{ArO}^\bullet) + H\,(\mathrm{H}^\bullet) - H\,(\mathrm{ArOH})$$
$$\mathrm{DPE} = H\,(\mathrm{ArO}^-) + H\,(\mathrm{H}^+) - H\,(\mathrm{ArOH})$$
$$\Delta G = G\,(\mathrm{product}) - G\,(\mathrm{reactants})$$

where *H* and *G* are the enthalpy and Gibbs free energy value at 298 K, and the calculated $H\,(\mathrm{H}^\bullet)$ or $G\,(\mathrm{H}^\bullet)$ in methanol was obtained from the literature [8]. The *H* and *G* were calculated as the sum of the thermal corrections and the single-point electronic energies.

In order to eliminate the influence caused by diffuse functions in some cases, the single-point energy files were regenerated at B3LYP-D3(BJ)/6–311G** level. Based on the files, the wave functions were analyzed *via* Multiwfn 3.8 program to obtain Hirshfeld charges of atoms and spin density of radicals [9]. The natural resonance theory (NRT) analysis was performed using NBO 7.0 program [10, 11]. All output files were visualized using GaussView 6.0, VMD 1.9.3, and Multiwfn 3.8 programs [9, 12, 13].

References

[1] Schmidt, B.; Riemer, M. Suzuki-Miyaura coupling of halophenols and phenol boronic acids: systematic investigation of positional isomer effects and conclusions for the synthesis of phytoalexins from pyrinae. *J Org Chem*. **2014**, 79, 4104-4118.

[2] Snyder, S.A.; Gollner, A.; Chiriac, M.I. Regioselective reactions for programmable resveratrol oligomer synthesis. *Nature*. **2011**, 474, 461-466.

[3] Denton, R.M.; Scragg, J.T.; Galofré, A.M.; Gui, X.; Lewis, W. A concise synthesis of honokiol. *Tetrahedron*. **2010**, 66, 8029-8035.

[4] Lee, D.; Hwang, W.; Choi, J.Y.; Ahn, I.; Lee, C.H. A convenient preparation of dityrosine via Mn(III)-mediated oxidation of tyrosine. *Process Biochem.* **2008**, 43, 999–1003.

[5] J.H., G., *Mass spectrometry*. 2013, Beijing: Science press.

[6] Frisch, M.; Trucks, G.; Schlegel, H.; Scuseria, G.; Robb, M.; Cheeseman, J.; Scalmani, G.; Barone, V.; Petersson, G.; Nakatsuji. Gaussian 16 Revision C. 01 (2016). *Gaussian Inc. Wallingford CT*. **2016**,

[7] Grimme, S.; Ehrlich, S.; Goerigk, L. Effect of the damping function in dispersion corrected density functional theory. *Journal of computational chemistry*. **2011**, 32, 1456-1465.

[8] Zheng, Y.-Z.; Chen, D.-F.; Deng, G.; Guo, R.; Fu, Z.-M. The antioxidative activity of piceatannol and its different derivatives: Antioxidative mechanism analysis. *Phytochemistry*. **2018**, 156, 184-192.

[9] Tian, L.; Chen, F. Multiwfn: A multifunctional wavefunction analyzer. *Journal of Computational Chemistry*. **2012**, 33, 580-592.

[10] Glendening, E.D.; Landis, C.R.; Weinhold, F. NBO 7.0: New vistas in localized and delocalized chemical bonding theory. *Journal of Computational Chemistry*. **2019**, 40,

[11] Glendening, E.D.; Weinhold, F. Natural resonance theory: I. General formalism. *Journal of Computational Chemistry*. **2015**, 19, 593-609.

[12] Dennington, R.; Keith, T.A.; Millam, J.M. GaussView 6.0. 16. *Semichem Inc.: Shawnee Mission, KS, USA*. **2016**,

[13] Humphrey, W.; Dalke, A.; Schulten, K. VMD: visual molecular dynamics. *Journal of molecular graphics*. **1996**, 14, 33-38.
