# Supplementary material for: Phytophenol Dimerization Reaction: From Basic Rules to Diastereoselectivity and Beyond
Source: Molecules. 2022 Jul 28;27(15):4842. doi: 10.3390/molecules27154842 (PMC9369853; doi:10.3390/molecules27154842)
Supplement: Supplementary file 1 [file molecules-27-04842-s001.zip › Supplementary File S3 45 instances of 3,7-dioxabicyclo[3.3.pdf]

Suppl. 3 45 instances of 3,7-dioxabicyclo[3.3.0]octane lignin with *trans*-αH,βH [1] [2] [3]

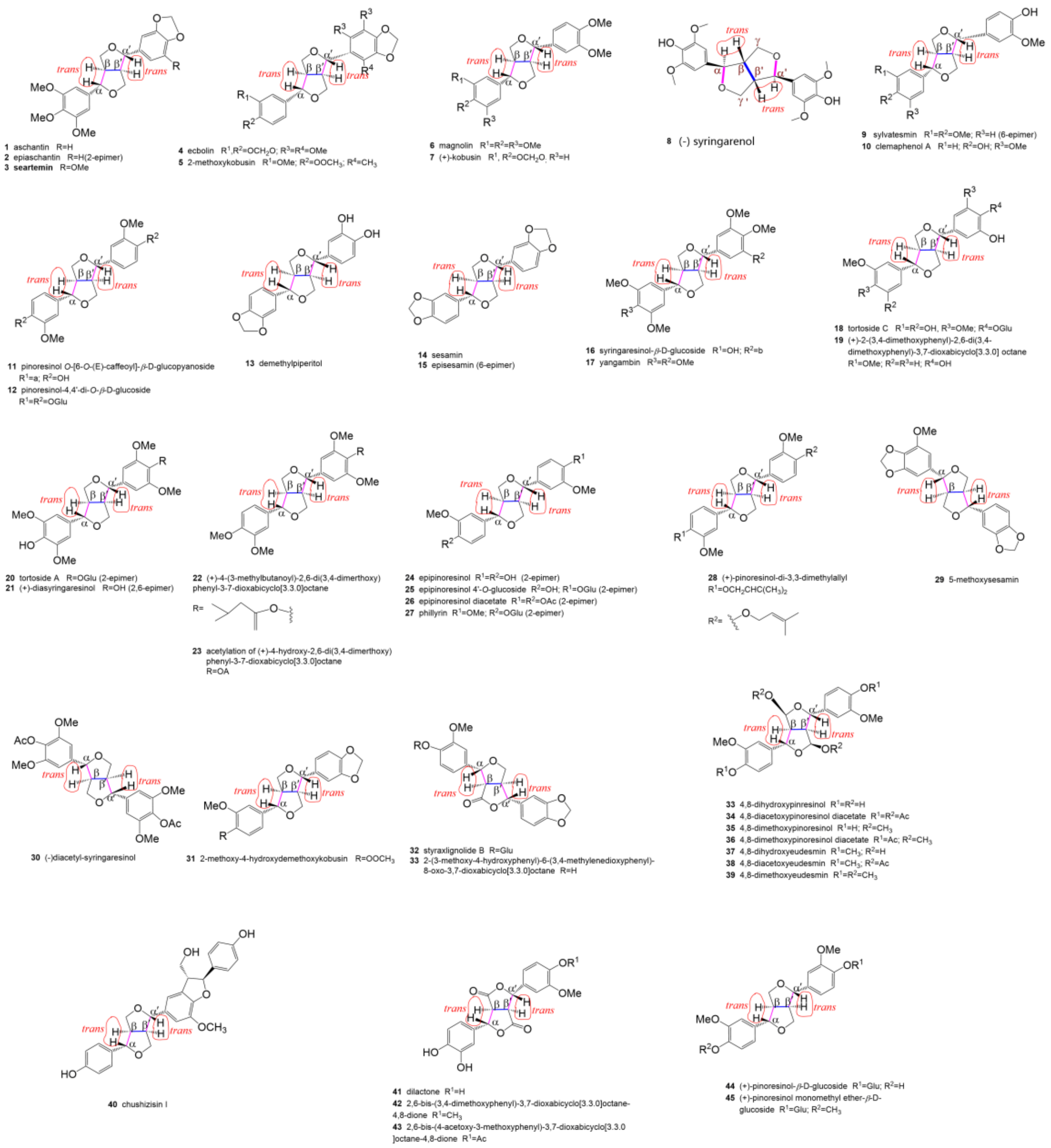

References

[1] Yang, J.S., *^{13}C-NMR Spectroscopic Databook of Natural Products*. stilbene. Vol. stilbene. 2011, Beijing: Chemical Industry Press. 2244-2277.

[2] Davin, L.B.; Wang, H.B.; Crowell, A.L.; Bedgar, D.L.; Martin, D.M.; Sarkanen, S.; Lewis, N.G. Stereoselective bimolecular phenoxy radical coupling by an auxiliary (dirigent) protein without an active center. *Science*. **1997**, 275, 362-366.

[3] Wang, C.; Tallian, C.; Su, J.; Vielnascher, R.; Silva, C.; Cavaco-Paulo, A.; Guebitz, G.M.; Fu, J. Ultrasound-assisted extraction of hemicellulose and phenolic compounds from bamboo bast fiber powder. *PLoS One*. **2018**, 13, e0197537.
