# Supplementary material for: Phytophenol Dimerization Reaction: From Basic Rules to Diastereoselectivity and Beyond
Source: Molecules. 2022 Jul 28;27(15):4842. doi: 10.3390/molecules27154842 (PMC9369853; doi:10.3390/molecules27154842)
Supplement: Supplementary file 1 [file molecules-27-04842-s001.zip › Supplementary File S4 Explanation or prediction in other fields.pdf]

Entry	Typical reaction equation	Explanations or prediction	Field
Eq. 1[1]	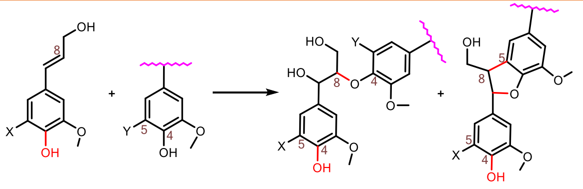	The exocyclic C=C involved PRC reaction also occurs in enzymatic lignification.[2],[3] Adhering to the second precondition, subunits in lignin bear *para*-OH groups. However, these subunits include caffeoyl, *para*-coumaroyl, feruloyl, and sinapyl.[4],[5],[6],[7] Occasionally, the *para*-OH group may also be expanded via conjugation or substitution. Eq. 1 is a typical instance. By comparison, there is neither *meta*-coumaroyl nor isoferuloyl because of the presence of a *meta*-OH group and the absence of *para*-OH group.[8, 9],[2, 7, 10], [11], [3]	lignin chemistry
Eq. 2[12]	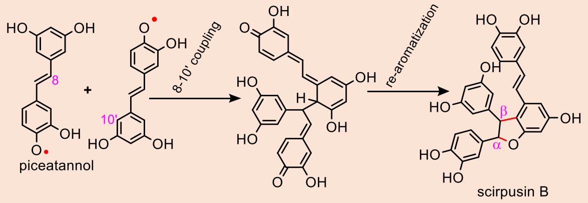	Eq. 2 shows the dimerization piceatannol to form scirpusin B. This is an exocyclic C=C participated PRC. Thus, piceatannol was found to co-exist with its furan-fused dimer, scirpusin B, the same plant as passion fruit seed.[13] Previous studies have not assigned the configurations of the two chiral atoms.[12],[13] Herein, the configuration of scirpusin B was predicted to be *αR,βR*, according to the aforementioned stereochemical characteristics.	
Eq. 3[14]	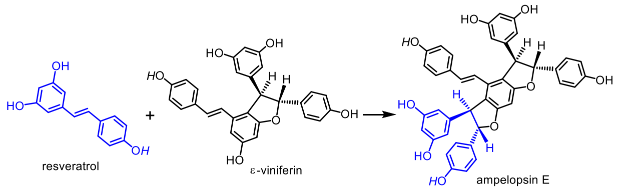	Eq. 3 describes the cross-dimerization of *E*-resveratrol monomer and its dimer, *trans* ε-viniferin. Thus, the product ampelopsin E can be considered as a trimer of *E*-resveratrol. The trimerization process is also controlled by relevant rules.	natural product chemistry
Eq. 4[15]	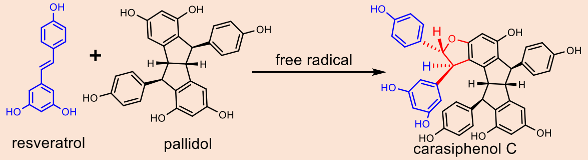	As mentioned above, the trimerization can be regarded as a PRC reaction between the monomer and dimer. Thus, stilbene trimerization complies with the rules, especially in terms of the stereoselectivity. This resembles the chemical synthesis of stilbene oligomers.[16]	

**Eq. 5**[17]	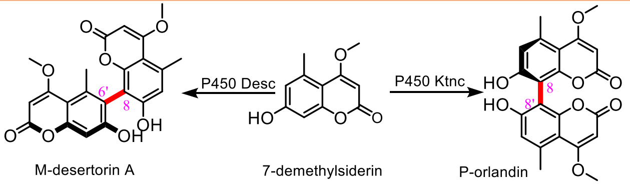	P450-mediated dimerization is a free radical process. According to the *meta*-excluded and C-C bonding domination rules, 7-demethylsiderin has three possible linkages, i.e., 8,8‘, 6,8‘, and 6,6‘. Our calculations indicate that these three possible linkages have similar energies (the ΔG values were calculated to be −1450.6955, −1450.6949, and −1450.6990 Hartree, respectively). Hence, any of these linkages are possible. However, the P450 enzyme excludes the 6,6‘-linkage.	synthetic chemistry
**Eq. 6**[18]	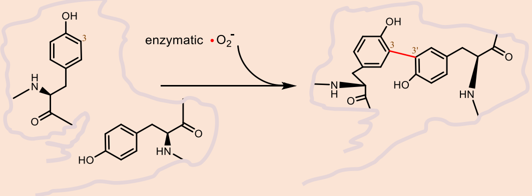	Cu^+ oxidation can lead to ROS formation. The ROS (such as $\bullet O_2^-$) can induce tyrosine to undergo a PRC reaction. According to the *meta*-excluded and C-C bonding domination rules, tyrosine can only use the 3-position for cross-linking.	enzyme chemistry
**Eq. 7**[19]	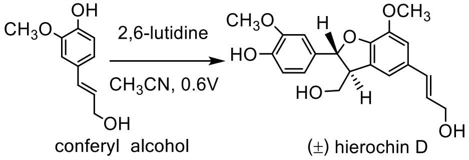	Eq. 7 is also an exocyclic C=C participated PRC reaction. However, the reaction is driven by current. Possibly due to the surplus energy, the reactant can overcome the energy barrier to produce a pair of enantiomers. However, no diastereomers are generated. This can also be attributed to the diastereoselectivity mentioned-above.	neurochemistry
**Eq. 8**[20]	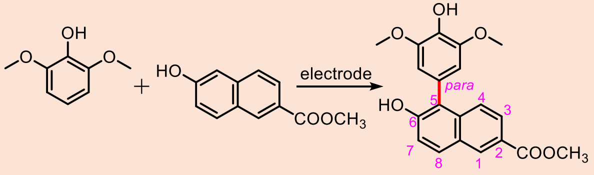	Under electrochemical conditions, the phenolic core can be oxidized into a radical intermediate and initiate a cross-PRC reaction. In line with the *meta*-excluded rule, both the 5- and 7-positions were preferential sites for reaction. Our calculations suggest that the ΔG value (−1224.0282 Hartree) of the *para*-5 linkage was lower than that of the *para*-7 linkage (−1224.0276 Hartree). Thus, the cross-PRC reaction can be described using Eq. 8.	electrochemistry

Eq. 9[21]

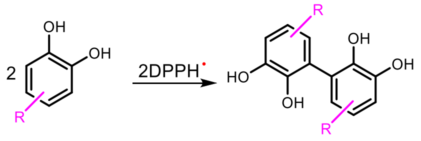

According to the *ortho*-diOHs co-activated rule, all sites may be linked and thus, the description shown in Eq. 9 is generally acceptable. A similar instance can also be found in the catecholic unit of peptides.[22]

References

1. van Parijs, F.R., et al., *Modeling lignin polymerization. I. Simulation model of dehydrogenation polymers.* Plant Physiol, 2010. **153**(3): p. 1332-44.
2. Sangha, A.K., et al., *Radical coupling reactions in lignin synthesis: a density functional theory study.* J Phys Chem B, 2012. **116**(16): p. 4760-8.
3. Voxeur, A., Y. Wang, and R. Sibout, *Lignification: different mechanisms for a versatile polymer.* Curr Opin Plant Biol, 2015. **23**: p. 83-90.
4. Petrik, D.L., et al., *p-Coumaroyl-CoA:monolignol transferase (PMT) acts specifically in the lignin biosynthetic pathway in Brachypodium distachyon.* Plant J, 2014. **77**(5): p. 713-26.
5. Obel, N. and H.V. Scheller, *Enzymatic synthesis and purification of caffeoyl-CoA, p-coumaroyl-CoA, and feruloyl-CoA.* Anal Biochem, 2000. **286**(1): p. 38-44.
6. Vismeh, R., et al., *Profiling of diferulates (plant cell wall cross-linkers) using ultrahigh-performance liquid chromatography-tandem mass spectrometry.* Analyst, 2013. **138**(21): p. 6683-92.
7. Zhu, X., et al., *Lignin-Biosynthetic Study: Reactivity of Quinone Methides in the Diastereopreferential Formation of p-Hydroxyphenyl- and Guaiacyl-Type beta- O-4 Structures.* J Agric Food Chem, 2019. **67**(8): p. 2139-2147.
8. Tobimatsu, Y. and M. Schuetz, *Lignin polymerization: how do plants manage the chemistry so well?* Curr Opin Biotechnol, 2019. **56**: p. 75-81.
9. Davin, L.B., et al., *Dissection of lignin macromolecular configuration and assembly: comparison to related biochemical processes in allyl/propenyl phenol and lignan biosynthesis.* Nat Prod Rep, 2008. **25**(6): p. 1015-90.
10. Morreel, K., et al., *Mass spectrometry-based sequencing of lignin oligomers.* Plant Physiol, 2010. **153**(4): p. 1464-78.
11. Reale, S., et al., *Lignin chemistry: biosynthetic study and structural characterisation of coniferyl alcohol oligomers formed in vitro in a micellar environment.* Chemistry, 2010. **16**(20): p. 6077-87.
12. Carlos Del Rio, J., et al., *Hydroxystilbenes Are Monomers in Palm Fruit Endocarp Lignins.* Plant Physiol, 2017. **174**(4): p. 2072-2082.
13. Sano, S., et al., *Identification of the strong vasorelaxing substance scirpusin B, a dimer of piceatannol, from passion fruit (Passiflora edulis) seeds.* J Agric Food Chem, 2011. **59**(11): p. 6209-13.

14. Wibowo, A., et al., *Malaysianol A, a new trimer resveratrol oligomer from the stem bark of Dryobalanops aromatica.* Fitoterapia, 2011. **82**(4): p. 676-81.
15. Snyder, S.A., A. Gollner, and M.I. Chiriac, *Regioselective reactions for programmable resveratrol oligomer synthesis.* Nature, 2011. **474**(7352): p. 461-6.
16. Keylor, M.H., et al., *Synthesis of resveratrol tetramers via a stereoconvergent radical equilibrium.* Science, 2016. **354**(6317): p. 1260-1265.
17. Mazzaferro, L.S., et al., *Cytochrome P450-catalyzed regio- and stereoselective phenol coupling of fungal natural products.* J Am Chem Soc, 2015. **137**(38): p. 12289-95.
18. Abeyawardhane, D.L., et al., *Copper Induced Radical Dimerization of alpha-Synuclein Requires Histidine.* J Am Chem Soc, 2018. **140**(49): p. 17086-17094.
19. Romero, K.J., et al., *Electrochemical Dimerization of Phenylpropenoids and the Surprising Antioxidant Activity of the Resultant Quinone Methide Dimers.* Angew Chem Int Ed Engl, 2018. **57**(52): p. 17125-17129.
20. Schafer, H.J., *Unsymmetrical Biaryl Compounds: Metal- and Reagent-Free Electrochemical Couplings Are on the Advance.* Angew Chem Int Ed Engl, 2017. **56**(49): p. 15502-15503.
21. Guitard, R., et al., *Myricetin, rosmarinic and carnosic acids as superior natural antioxidant alternatives to alpha-tocopherol for the preservation of omega-3 oils.* Food Chem, 2016. **213**: p. 284-295.
22. Lampel, A., et al., *Polymeric peptide pigments with sequence-encoded properties.* Science, 2017. **356**(6342): p. 1064-1068.
